# Effects of genetic polymorphism of drug-metabolizing enzymes on the plasma concentrations of antiepileptic drugs in Chinese population

**DOI:** 10.1080/21655979.2022.2036916

**Published:** 2022-03-15

**Authors:** Weixuan Zhao, Hongmei Meng

**Affiliations:** Department of Neurology, the First Hospital of Jilin University, Jilin University, Changchun, China

**Keywords:** Genetic polymorphism, antiepileptic drug, plasma concentration, valproic acid, carbamazepine, phenytoin, levetiracetam, lamotrigine, oxcarbazepine

## Abstract

As a chronic brain disease, epilepsy affects ~50 million people worldwide. The traditional antiepileptic drugs (AEDs) are widely applied but showing various problems. Although the new AEDs have partially solved the problems of traditional AEDs, the current clinical application of traditional AEDs are not completely replaced by new drugs, particularly due to the large individual differences in drug plasma concentrations and narrow therapeutic windows among patients. Therefore, it is still clinically important to continue to treat patients using traditional AEDs with individualized therapeutic plans. To date, our understanding of the molecular and genetic mechanisms regulating plasma concentrations of AEDs has advanced rapidly, expanding the knowledge on the effects of genetic polymorphisms of genes encoding drug-metabolizing enzymes on the plasma concentrations of AEDs. It is increasingly imperative to summarize and conceptualize the clinical significance of recent studies on individualized therapeutic regimens. In this review, we extensively summarize the critical effects of genetic polymorphisms of genes encoding drug-metabolizing enzymes on the plasma concentrations of several commonly used AEDs as well as the clinical significance of testing genotypes related to drug metabolism on individualized drug dosage. Our review provides solid experimental evidence and clinical guidance for the therapeutic applications of these AEDs.

## Graphical Abstract

**Figure:**
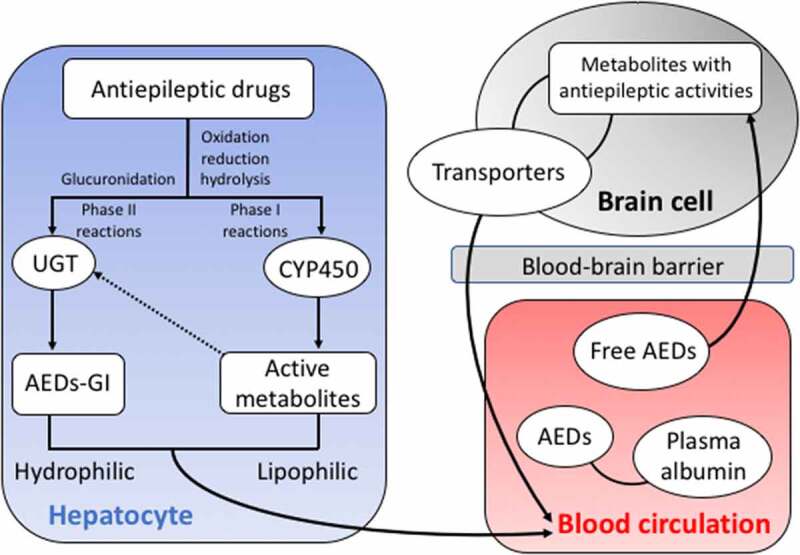

## 1. Introduction

Epilepsy is a group of chronic brain diseases in humans characterized by transient dysfunctions in the central nervous system caused by abnormal discharge of brain neurons, generally happening unexpectedly and repeatedly. Approximately 50 million people are affected worldwide and nearly 80% of patients can completely control their seizures and restore their normal quality of life through medication [1]. The antiepileptic drugs (AEDs) function by directly inhibiting the excessive discharge of neurons in the lesion and acting on the normal brain tissue around the lesion to prevent the spread of abnormal discharge of brain neurons [2]. The AEDs function following several known mechanisms [3]. First, the AEDs block the sodium channels. By acting on voltage-dependent sodium channels, the frequency of continuous action potentials in the neurons is reduced. This effect is further enhanced during depolarization. It is now well-known that repeated depolarization is an important mechanism underlying seizures of epilepsy. Second, the AEDs enhance the bioactivities of γ-aminobutyric acid (GABA) in various ways to ultimately inhibit the high-frequency repetitive action potentials. Third, the AEDs antagonize excitatory amino acids, which, together with their receptors, are involved in the occurrence of epilepsy. It has been well demonstrated in a variety of animal models of epilepsy that the seizures of epilepsy are inhibited by reducing the activities of excitatory transmitters (*i.e*., glutamate) in different ways.

The old generation AEDs, *e.g*., carbamazepine (CBZ), phenytoin (PHT), valproic acid (VPA), and phenobarbital (PB), have been widely used in clinical settings, even though they have shown serious problems, including the nonlinear pharmacokinetics, large individual differences, narrow treatment ranges, severe adverse reactions, and drug interactions. The new generation AEDs that have been introduced in the past 10 years, such as levetiracetam (LEV), lamotrigine (LTG), and oxcarbazepine (OXC), have shown fewer adverse reactions, higher bioavailability, and less drug interactions, partially solving the problems of the traditional AEDs. However, the current clinical applications of traditional AEDs cannot be completely replaced by the new drugs, particularly due to the large individual differences in plasma concentrations of AEDs and narrow therapeutic windows, which are the important measurements in clinical treatment of epilepsy.

In recent years, significant progress has been made to explore the genetic mechanisms affecting the plasma concentrations of AEDs [3]. The relevant genes have been extensively investigated and reviewed in various ethnic groups worldwide [4–12]. Furthermore, studies have showne that the genetic polymorphisms of these relevant genes also play important roles in the diagnosis of epilepsy. For example, Li *et al*. [13] have investigated the functions of TUG1 in restoring hippocampal neuronal activities and preventing neuronal cell death. Their results show that silence of TUG1 increases the viability of hippocampal neurons and inhibits their apoptosis. The diagnostic capability of the elevated levels of TUG1 measured in children with temporal lobe epilepsy (TLE) has been further explored, revealing that TUG1 shows high sensitivity and specificity in children with TLE, strongly suggesting the diagnostic potential of TUG1 in children with TLE. Ahmed *et al*. [14] have investigated the genetic polymorphisms of *CYP2C19* in the healthy individuals of six distinct ethnic populations of Pakistan. The results show that the minor allele is present at a frequency of 15.06% and 8.14% for CYP2C19*2 and CYP2C19*3, respectively, suggesting that a significant portion of the Pakistani population and its certain ethnic groups contain high frequencies of polymorphisms with reduced functions. However, a comprehensive review of studies in this area is still lacking in Chinese populations. In this review, we first summarize the main factors affecting the metabolism of six types of AEDs (*i.e*., three traditional AEDs including VPA, CBZ, and PHT and three new AEDs including LEV, OXC, and LTG) currently commonly used in treating patients of epilepsy, including their general metabolic characteristics. We further analyze the effects of the genetic polymorphisms of genes encoding the metabolic enzymes on the plasma concentrations of AEDs and the clinical significance of the drug metabolism-related genotype testing on individualized drug dosage. We strongly believe that this comprehensive review on most of AEDs currently popularly used in clinical practice provides strong evidence and clinical guidance in the therapeutic applications of these AEDs.

## 2. Pharmacokinetic characteristics of traditional antiepileptic drugs

**Figure 1.**
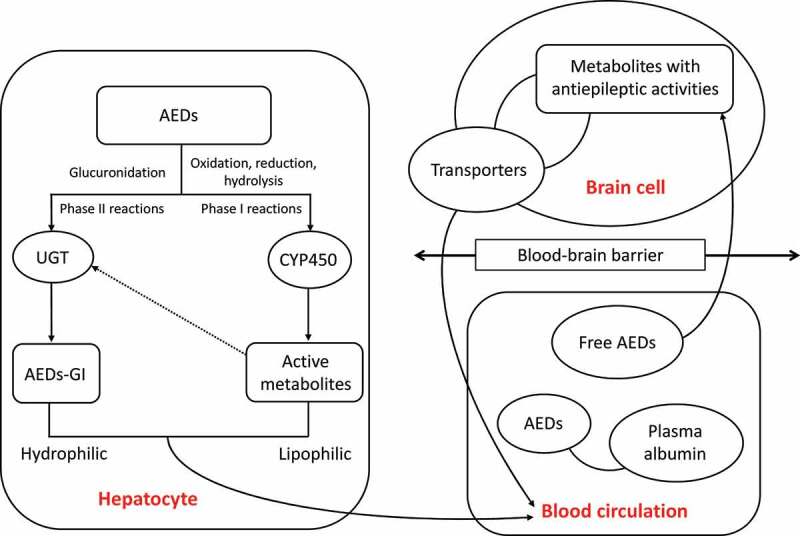
Schematic pathways of antiepileptic drug (AED) metabolizing enzymes in both liver and brain cells and the blood circulation system. The arrowed dashed line indicates the participation in phase II reactions of a portion of the active metabolites derived in phase I reactions. AEDs-GI represents the glucuronidation products of AEDs.

The metabolism of traditional AEDs in the liver includes phase I and phase II reactions (Figure 1). With a large amount of exogenous substances undergoing biological transformations in the body, the liver is the main location for the phase I metabolic reactions of the biological transformation. During the phase I reaction, the drugs undergo oxidation, reduction, and hydrolysis catalyzed by the monooxygenase system with cytochrome P450 (CYP450) as the core to change the molecular structure, to increase the polarity of the drugs and the water solubility, ultimately to change the activities and functions of the drugs. The phase I metabolic reactions are the rate-limiting steps for the elimination of drugs from the body, causing either detoxification or poisoning effects [15]. A portion of the active metabolities derived in phase I reactions enters the phase II reactions. During the phase II reaction, the drug and its metabolites combined with endogenous substances are excreted from the body, showing a detoxification effect. However, some active metabolites generated simultaneously during the phase II reactions probably cause the liver damage [16]. The uridine diphosphate glucuronidase (UGT) is the main metabolic enzyme catalyzing the binding reaction during phase II reaction [17]. Following the phase I and II reactions, the AEDs are absorbed into the blood and partially combined with plasma albumin. Then, the AEDs penetrate the blood-brain barrier and are eventually distributed in the brain, while the drug transporters (*i.e*., the functional membrane proteins located on the cell membrane) mediate the drugs to enter the cells by taking up the substrate to the target site to exert its effect and to efflux the drugs out of the cells thus reducing the plasma concentrations and drug efficacy.

We here review the studies on the relationships between the plasma concentrations of these traditional AEDs (i.e., VPA, CBZ, and PHT) and the genetic polymorphisms of genes encoding enzymes involved in the metabolism of these AEDs.

## 3. Valproic acid

### 3.1. Metabolic characteristics of valproic acid

As a type of broad-spectrum AEDs, the valproic acid (VPA) has been widely used in clinical applications. VPA shows antiepileptic effects by inhibiting the sodium ion channels and possibly the T-type calcium ion channels, inhibiting the metabolic enzymes GABA to cause the accumulation of GABA in brain, and inhibiting excessive discharge of neurons at the lesion and simultaneously preventing the spread of abnormal discharge of brain neurons [18]. The effective plasma concentrations of VPA range from 50 to 100 ug/ml.

The pharmacokinetics of VPA conforms to the one-compartment model and the first-order kinetic process. The oral absorption of VPA is rapidly distributed in the body. Generally, the highest plasma concentration is obtained in 0.5–2 hours after the medicine is taken with an empty stomach or 2–4 hours after taken with food. The effective threshold of VPA varies among different patients with 50–100 ug/ml for 50% of patients, 25–50 ug/ml for 20% of patients, while 10% of patients have a range of either 100–150 ug/ml, 10–25 ug/ml, or less than 10 ug/ml [19]. It is generally believed that the toxic reactions would be caused by the plasma concentrations over 100 ug/ml, while clinical improvement of symptoms is observed with the drug plasma concentrations of 50–60 ug/ml.

After the VPA is absorbed into the blood, it is mainly combined with plasma albumin with a binding rate of 84%-94%, while the concentration of free VPA is generally low. Different patients show different plasma albumin binding rates with VPA, probably due to the fatty acids and applications of other medicines with high binding capability with the plasma albumin. VPA can easily penetrate the blood-brain barrier and is evenly distributed in the brain. VPA can also pass through the placental barrier to cause teratogenic effects on the fetus, such as the spina bifida [20]. VPA shows a slightly higher rate of teratogeny than those of other tranditional AEDs, *e.g*., CBZ and PHT.

### 3.2. Effect of cytochrome P450 on the metabolism of valproic acid

As a group of monooxygenases, the cytochrome P450 (CYP450) enzyme system contains enzymes that mainly exist in the microsomes of liver cells and are located on the endoplasmic reticulum membranes. These enzymes are a group of structurally and functionally related isozymes involved in the metabolism of many endogenous and exogenous substances (i.e., the detoxification of exogenous substances and activation of pre-carcinogens) and encoded by genes in the CYP superfamily containing more than 10 families of over 100 genes [21,22]. There are only three groups of genes (*i.e., CYP1, CYP2*, and *CYP3*) involved in regulating the metabolism of most drugs and foreign substances with CYP2 as the largest family containing 15 subfamilies (CYP2A to 2Q), metabolizing 40% of clinical drugs currently prescribed. It is estimated that about 56% of drugs are mainly metabolized by CYP450 enzymes, with CYP3A4 involved in metabolism of more than 50% of human drugs, followed by CYP2D6 (30%), CYP2C9 and CYP2C19 (12%), CYP1A2 (4%), CYP2E1 (2%), and CYP2A6 (2%) [23,24].

We here review the recent advancement in our understanding of the functions of these CYP450 enzymes in the metabolism of VPA. Studies have shown that four enzymes (*i.e*., CYP2A6, CYP2B6, CYP2C9, and CYP2C19) are the main metabolizing enzymes of VPA with their metabolic activities closely related to the metabolism of VPA [25]. The wild types of these genes show significantly stronger metabolizing capability on VPA than the mutants. Patients carrying the mutant *CYP* genes show decreased capability of removing VPA, causing the accumulation of VPA in the body to induce adverse reactions, such as decreased carnitine, increased blood ammonia, and abnormal blood index [26]. Furthermore, studies have also confirmed that the hepatotoxic metabolites of VPA are mainly produced by the CYP pathway [27,28]. Therefore, the genotypes encoding the CYP450 enzymes play important regulatory roles in maintaining the level of VPA in patients.

#### 3.2.1. CYP2A6

The enzymatic activity of CYP2A6 regulates the metabolic rate of sodium valproate *in vivo*. The *CYP2A6* gene contains the alleles of wild-type CYP2A6*1 and mutant CYP2A6*4 [29]. The generation of CYP2A6*4 is caused by the deletion of *CYP2A6* gene. Oscarson *et al*. have shown that the activity of CYP2A6 with *4 allele is reduced or even lost completely [30]. Furthermore, Several studies have reported that the allele frequency of CYP2A6*4 is 13.3–15.1% in the Chinese populations [30–32]. Sun *et al*. have reported that the average standard plasma concentration of sodium valproate in patients with CYP2A6*4 allele is significantly higher than that of the patients without CYP2A6*4 allele, suggesting that the polymorphism of *CYP2A6* gene affects the plasma concentration of sodium valproate [31]. The patients with CYP2A6*4/*4 genotype generate inactivated enzyme due to the complete deletion of the *CYP2A6* gene [30], while genotype CYP2A6*1/*4 generates enzyme with reduced activity due to having one copy of wild type *CYP2A6* allele, indicating that the plasma concentration of sodium valproate in patients with *4 allele is significantly higher than that of patients without *4 allele [30]. This study also showed that when given regular doses, the plasma concentration of the medicine in patients with CYP2A6*4 allele was higher than that of the patients without CYP2A6*4. Therefore, it is highly recommended that patients with CYP2A6*4 allele should be clinically treated with reduced dosage of sodium valproate and monitored closely for the occurrence of adverse reactions.

#### 3.2.2. CYP2B6

CYP2B6 is the main member of the CYP2B subfamily and is the only homologous protein with enzymatic activity in this subfamily of humans. The *CYP2B6* gene has been mapped to the location 19q12 ~ 19q13.2 on chromosome 19, containing 9 exons and 6 alleles with 9 mutation sites identified [32–34]. As one of the 6 alleles, CYP2B6*6 contains two mutation sites, *i.e*., 516 G > T on exon 4 and 718A>G on exon 5, with the mutation rates of 21% and 28%, respectively, in the Chinese Han population [35,36]. Using the gene point mutation detection approach, Hideto *et al*. [37] reported that at the Lys262Arg site, amino acid substitutions were observed in three enzymes (*i.e*., encoded by CYP26B*4, CYP26B*6, and CYP2B6*7), showing higher Vmax and Vmax/Km values than those of the non-mutant type. These results indicate that the catalytic activity of the CYP2B6 enzyme coded by the mutated *CYP2B6* genotype has been significantly altered [37], which is probably the main molecular mechanism generating the variations in the metabolic functions of each genotype of *CYP2B6* on the heterogeneous compounds [38]. Tan *et al*. have reported that the average plasma concentration of VPA in patients with CYP2B6*6 allele is higher than that of wild-type patients (*i.e*., homozygous for CYP2B6*1), while the metabolism of VPA slows down in the group with mutations of *CYP2B6* gene [39]. Therefore, it is speculated that the genetic polymorphism of *CYP2B6* affects the plasma concentration of VPA, while the metabolism of VPA is regulated by *CYP2B6* gene. Furthermore, studies have shown that patients with CYP2B6*1/*6 genotype show higher average drug plasma concentrations than patients with CYP2B6*1/*1 genotype, suggesting that patients with mutant genes may lose the enzymatic activity due to the lack of the gene, decrease the VPA metabolism, and keep the drug plasma concentration relatively high [39]. These studies indicate that in the clinical application of VPA, in order to avoid the VPA poisoning caused by excessive drug concentration, patients with mutant *CYP2B6* gene are initially treated with a small dose to achieve effective drug plasma concentration in order to control the symptoms [37–39]. This strategy could reduce the waste of medicinal resources and, more importantly, prevent the adverse reactions of medicines and possible serious consequences. Therefore, it is recommended that when treating patients with mutant *CYP2B6* genes, clinicians should follow the principle of starting with a small dose, while closely observing symptoms and monitoring changes in drug plasma concentration in patients.

#### 3.2.3. CYP2C9 and CYP2C19

CYP2C is the main component of CYP450, accounting for 20% of the entire family of CYP450. Both *in vitro* and *in vivo* studies have shown that both CYP2C9 and CYP2C19 are the key metabolic enzymes for the biological transformation of VPA [40]. CYP2C9 is responsible for most (~75-80%) of VPA terminal desaturation and hydroxylation reactions and closely related to the generation of the hepatotoxic products of VPA [41]. CYP2C9*3 is a common polymorphic site of *CYP2C9* allele in Chinese Han population. The A1075C site of CYP2C9*3 is the main mutation site in the Chinese population with the allele frequency of ~3.3% [42]. The two common polymorphic sites of *CYP2C19* allele are CYP2C19*2 (G681A; rs4244285) and CYP2C19*3 (rs4986893, c.636 G > A). Studies have shown that CYP2C19*2 causes the splicing mutation inactivation of the transcription protein, while CYP2C19*3 forms a terminator to disrupt the activity of the transcription proteins, causing decreased enzymatic activity to metabolize VPA, ultimately increasing the drug plasma concentration and causing side effects related to plasma concentration [43].

Among the known polymorphic sites of *CYP2C9* gene, the CYP2C9*3 mutation is commonly detected in the Chinese population, while the mutation of CYP2C9*2 is rarely reported [44]. Studies have shown that the metabolites of VPA include 4-Ene-VPA, 4-OH-VPA, and 5-OH-VPA, with 4-Ene-VPA confirmed to show liver toxicity [45]. In the *CYP2C9* heterozygous mutant, the production of 4-Ene-VPA is 29% less than that of the wild homozygotes, while the mutant homozygotes generate 61% less of 4-Ene-VPA compared to wild homozygotes, indicating that patients with wild-type CYP2C9*1 are more likely to show liver toxicity than the carriers of CYP2C9*3.

Studies have shown that the plasma concentration of VPA in AA genotype at CYP2C19*2 locus is significantly higher than that of the GG genotype after the medicine is taken for 2, 4, and 8 hours [46], while the plasma concentration of VPA in AA genotype at CYP2C19*3 locus is significantly higher than that of the GG genotype 8 hours after the medicine is taken [47]. No significant difference is detected in the plasma concentration of VPA among other genotypes. The allele frequencies of CYP2C19*2 mutant (AA type) and CYP2C19*3 mutant (GG type) in Chinese population are 37.16% and 14.19%, respectively. These studies demonstrate that the genetic polymorphism of CYP2C19*2 gene is closely related to the plasma concentration of VPA in patients with epilepsy, ultimately affecting the therapeutic effect of VPA. These results are consistent with those reported previously [48,49]. Therefore, it is recommended that when VPA is used in the treatment of epilepsy, the dosage should be increased for patients with CYP2C19*3 GG genotype showing fast metabolism, while the dosage be appropriately reduced for patients with CYP2C19*3 AA genotype.

Using a combined analysis based on the mutant alleles of both *CYP2C9* and *CYP2C19* genes, Tan *et al*. categorized patients of epilepsy into three groups, including (1) extensive metabolizers (EM) with the wild-type genotypes of the homozygous combination of CYP2C9*1*1 and CYP2C19*1*1, (2) intermediate metabolizers (IM) with the heterozygous combinations of two out three genotypes (CYP2C9*1*3, CYP2C19*1*2, or CYP2C19*1*3), and (3) poor metabolizers (PM) with genotypes of mutant homozygous combination of either CYP2C9*3*3, CYP2C9*1*3, or CYP2C19*2*2 [50]. The frequencies of the three groups of patients are 47.5%, 25%, and 27.5%, respectively. The number of mutant genes is positively correlated with the standardized plasma concentration, while the plasma concentration of PM patients is significantly higher than that of EM patients with equal dose of VPA taken.

These studies conclude that both CYP2C9 and CYP2C19 are involved in the metabolism of VPA in humans with their genetic polymorphisms significantly affecting the metabolic process of VPA [45–50]. Therefore, in clinical treatments, it is substantially important to detect the genotype of patients in order to establish precise formulation of individualized drug dosages and to effectively avoid drug waste and adverse reactions, exhibiting significant clinical importance for individualized drug application.

#### 3.2.4. CYP2D6

The gene encoding CYP2D6 is located in region 1 (*i.e*., band 3 and subband 1) on the long arm of chromosome 22 in humans, playing important roles in genetic pharmacology. Studies have shown that CYP2D6 is clinically involved in the metabolism of more than 25% of commonly used drugs, such as isoquinoline (adrenergic blocking drug), spapartin and propafenone (antiarrhythmic drugs), and amitriptyline (antidepressants) [51]. In recent years, the distribution of *CYP2D6* genetic polymorphisms in different human populations and its relationship with the effects of clinical therapeutic drugs have been widely explored [52,53]. The frequency of CYP2D6*10 allele is relatively high in Chinese population (50–70%), affecting the safety and effectiveness of substrate drugs in clinical applications.

By comparing the plasma concentration of sodium valproate and standardized drug plasma concentration in patients with epilepsy of different genotypes of CYP2D6*10, studies have reported that the drug plasma concentrations of three genotypes of CYP2D6*10 are not significantly different from the standardized drug plasma concentration [54]. Specifically, patients of epilepsy with different genotypes of CYP2D6*10 not reaching the minimum effective plasma concentration and those exceeding the minimum toxic concentration are compared to investigate their genetic polymorphisms. The results show that there is no significant difference between the number of CYP2D6*10 genotypes not reaching the minimum effective concentration and the number of cases exceeding the minimum toxic concentration, indicating that the genetic polymorphism of CYP2D6*10 is not involved in the regulation of the plasma concentration of sodium valproate in patients with epilepsy.

### 3.3. Effect of uridine diphosphate glucuronidase on the plasma concentration of valproic acid

The uridine diphosphate glucuronidase (UGT) is widely distributed in many organs and tissues in humans such as liver, kidney, heart, brain, and skin, with the liver showing the highest activity [55,56]. UGT is mainly expressed in the liver with a wide range of substrates, catalyzing the metabolic reactions of both endogenous substances (*i.e*., steroid hormones, bile acid, and tretinoin) and exogenous substances such as drugs (*i.e*., CBZ, valproate, non-steroidal anti-inflammatory drugs, morphine, zidovudine, chloramphenicol, and farnesol) [57]. As an important enzyme participating in the phase II metabolic reaction in the human body, UGT increases the water solubility of its metabolic substrates through glucuronidation, ultimately promoting the excretion of these substrates. Due to its polymorphic nature, the *UGT* gene encodes enzymes with varied metabolic efficiency and metabolic rates on their substrates [58]. In humans, the genes encoding UGT are mainly divided into two families, *UGT1* containing subfamily *UGT1A* and *UGT2* containing subfamilies *UGT2A* and *UGT2B* [59].

Studies have shown that compared with the effect of *CYP450* genetic polymorphism on the plasma concentration of VPA, the *UGT* genetic polymorphism shows larger impact (generally by >30% or up to 50% for some sites) on the plasma concentration of VPA than that of CYP450 [57–60]. In humans, a total of 19 active UGT subtypes have been identified with 7 subtypes (*i.e*., UGT1A3, UGT1A4, UGT1A6, UGT1A8, UGT1A9, UGT1A10, and UGT2B7) involved in the process of VPA glucuronidation, while UGT1A8 and UGT1A10 are expressed in extrahepatic tissues but rarely expressed inside the liver, showing trivial effect on the metabolism of VPA.

#### 3.3.1. UGT1A3

UGT1A3 is mainly expressed in the liver as well as the biliary and gastrointestinal tracts. The *UGT1A3* gene shows a high mutation rate with many mutation sites identified. To date, a total of 31 single nucleotide polymorphisms (SNPs) have been identified in the promoter region and the first exon of *UGT1A3* gene, caused mainly by alkaline substitutions [61].

Studies have shown that mutations are identified in four (*i.e., T31C, G81A, A477G*, and *A17G*) out 7 loci of *UGT1A3*, while no mutation is revealed in other three loci (*i.e., C133T, A808G*, and *G342A*) [61]. The standardized plasma concentration of sodium valproate in children with heterozygous mutations is significantly lower than that of the wild type homozygotes with only the *T31C*. With the high allele frequency (36.67%) of *T31C*, it is concluded that the *T31C* genetic polymorphism impacts greatly on the plasma concentration of sodium valproate in children with epilepsy, suggesting that children with heterozygous mutations should be treated with increased dosage of sodium valproate to reach the effective range of plasma concentration [61]. These conclusions are consistent with the results reported by Cho *et al*. [62] showing that the *UGT1A3* gene can affect the activity of its own transcription enzymes. The inconsistency of these results may be caused by the small sample size of the study and the difference in the characteristics of drug metabolism between adults and children. To date, studies on the effects of UGT1A3 on AEDs worldwide are still lacking and further studies are needed to investigate the roles that UGT1A3 plays in the alteration of drug plasma concentration.

#### 3.3.2. UGT1A6

UGT1A6 is distributed in the liver, gallbladder, colon, stomach, and brain. This enzyme is composed of 531 amino acids and exhibits catalytic activity in the form of either dimers or tetramers. To date, the studies on the effect of UGT1A6 on VPA glucuronidation are intensive and relatively highly advanced. Studies have shown that the *UGT1A6* gene contains a total of 28 known SNPs with 3 closely related but not completely linked sites, *i.e., T19G* (S7A), *A541 G* (T181A), and *A552C* (R184S), showing the highest mutation frequency and being extensively studied [61].

Studies by Kang *et al*. on the genetic polymorphisms of *UGT1A6 A541 G* and *A552*C genes show that the mutation rates of *A541 G* and *A552C* are 33.7% and 31.0% [63], respectively, which are slightly higher than 22.0% and 24.7%, respectively, as reported by Xing *et al*. [64]. Furthermore, Kang *et al*. have revealed no significant difference in the sodium valproate plasma concentration in patients with *UGTlA6 A541 G* genotype [63]. The same results are also reported by Wang *et al*. [65]. Jin *et al*. have documented that the *A541 G* mutation rate in patients with epilepsy in Chinese Han populations is 28.9% [66]. Results of statistical analysis show that the plasma concentration of VPA in patients with heterozygous *UGT1A6* mutant alleles is different from that of the wild-type patients. Most wild-type patients are normal or slow metabolizers with the measured drug plasma concentration consistent with the effective drug plasma concentration, while most mutant patients are fast metabolizers with the measured drug plasma concentration relatively lower than the effective drug plasma concentration. These results are consistent with those reported in previous studies [67]. Based on the statistical analysis of the mutation of *UGT1A6 A522C*, it is concluded that the gene mutation frequency of *UGT1A6 A522C* is 26.25% and the plasma concentration of VPA is significantly higher in the carriers of mutated gene, while the plasma concentration in patients with the homozygous mutation is higher than that of the heterozygous patients [68]. No statistical difference is revealed between the heterozygous and homozygous patients due to the small sample size of the homozygous carriers. These results are consistent with those reported by Kang *et al*. [63]. Furthermore, studies by Mei *et al*. show that mutations at *rs6759892* (*T19G*) and *rs1105879* (*A552C*) cause the increase of enzymatic activity, leading to decreased plasma concentration of VPA [69].

In summary, results based on various studies on each of the mutation sites of *UGT1A6* gene are generally consistent, suggesting that mutations of *UGT1A6* gene lead to enhanced enzymatic activity, accelerated VPA metabolism, and reduced drug plasma concentration [63–69]. Therefore, it is recommended that the dosage of medicines for patients with mutations at these common mutation sites of *UGT1A6* could be appropriately increased in order to quickly reach the therapeutic window and to optimize the therapeutic effect of the medicines.

#### 3.3.3. UGT1A9

UGT1A9 is mainly expressed in the liver (with relatively high expression level) and kidney, playing an important role in the metabolism of drugs and foreign substances. To date, a total of 21 alleles have been identified in the *UGT1A9* gene, including 11 located in the promoter, 4 in the first exon, and 6 in the first intron [61]. Guo *et al*. have reported that the 4 gene mutations of *UGT1A9* located in the first exon cause changes in metabolic capacity [70]. However, these mutations are rare in various ethnic groups with a frequency of less than 1%, showing trivial significance in population studies of these mutations. Similarly, Lin *et al*. have reported that based on the results of statistical analysis on multiple mutation sites of *UGT1A9*, no mutations are detected in *rs72551330* (*T98C*) in selected populations [71]. These results are consistent with the findings by Guo *et al*. [70], *i.e*., the *UGT1A9* mutations, including *rs13418420* (*T1819C), rs2741045* (*C441T), rs2741049* (*CI399T*), and *rs6731242* (*T1888G*), showing no significant effects on the plasma concentration of VPA.

#### 3.3.4. UGT2B7

UGT2B7 is mainly expressed in the liver and is the most important glucuronyl transferase with a wide range of substrates and the strongest activity in VPA metabolism. The *UGT2B7* gene shows a high degree of polymorphism with more than 30 mutations reported in the promoter, intron, and exon regions [61]. The results of a genetic study of *UGT2B7* based on 239 Norwegian patients by Holthel *et al*. [72] show that linked mutations are revealed in the *UGT2B7* promoter region at the sites 1246, 1239, 840, 268, 211, 161, and 102, while the genetic polymorphisms at the sites 268 and 102 affect the clearance efficacy of VPA.

Results of a statistical analysis by Zou *et al*. show that the mutation rate of *UGT2B7 A268G* in the Chinese Han population is 69.95% [73], which is similar to the mutation rate of 70.0% reported by Zhang *et al*. [74]. Both of these studies conclude that the genotypes of the patients are associated with the VPA plasma concentration. In particular, the plasma concentration in patients of wild-type homozygous is significantly higher than those of the other genotypes, while the difference in the average plasma concentration among patients of different genotypes is statistically significant. The mutation of the bases from A to G at this site leads to an increase in the ability of patients to metabolize VPA and a decrease in drug plasma concentration. Because the metabolic capacity of wild-type patients is relatively weak, these patients may be treated with reduced dosage of medicine or adjusted medication program.

Zou *et al*. have reported that the mutation frequency of *G211T* locus in the Chinese Han population is 22.58% [73], which is slightly higher than 15.8% as previously reported [75]. No significant difference is revealed in the plasma concentration of VPA among patients with different genotypes at this locus. The same results are also reported by Sun *et al*. [57]. Studies on the *UGT2B7 C802T* locus by Zhang *et al*. show that the mutation rate of the selected population is 63.73% [76], which is comparable to the 68.5% reported by Chu *et al*. [77]. The plasma concentrations of patients with both CT and TT genotypes are significantly lower than that of CC genotype, indicating that the mutation at *C802T* site leads to the enhanced enzymatic activity and increased VPA metabolism. These conclusions are supported by the results reported previously [78]. Therefore, it is recommended that the patients of epilepsy with mutant *UGT2B7 C802T* gene be given a relatively high dose of VPA for the initial treatment. Then, after reaching a steady state, the plasma concentration is monitored to achieve an effective plasma concentration. This therapeutic strategy is suggested to avoid insufficient drug concentration to affect its efficacy.

In the UGT family, the relationship between *UGT1A6* and *UGT2B7* genetic polymorphisms and their effects on the VPA plasma concentration of patients with epilepsy is relatively clear, while the data on other genetic subtypes are lacking and the possible linked mutations in other genotypes cannot be ruled out [79]. In order to study the overall effects of the genotypes on the plasma concentration of VPA, it is necessary to investigate not only the SNPs with prominent impact, but also the comprehensive analysis of multi-site polymorphisms [80]. As an important metabolic enzyme involved in phase II reaction, the genetic polymorphism of *UGT* inevitably plays an important role in the clinical drug application, providing imperative guidance for precise therapy of patients.

### 3.4. Effect of transporters on the plasma concentration of valproic acid

Drug transporters are a group of functional membrane proteins located on the cell membranes. There are many types of transporters involved in drug transmembrane transport in the human body. Based on the direction of substrate transmembrane transport, these transporters are divided into two major types, one type involved in drug absorption and the other in drug efflux [81]. The transporters mediating the drug to enter the cells take up the substrate to the target site to exert its effect. These transporters contain solute carriers (SLCs), mainly including L-type amino transporters (LATs), peptide transporters (PEPTs), sodium dependent secondary active transporters (SGLTs), sodium-independent facilitated diffusion transporters (GLUTs), monocarboxylate transporters (MCTs), organic anion transporters (OATs), and organic cation transporters (OCTs). Transporters mediating drug efflux mainly include P-glycoprotein (P-gp), multi-drug resistance associated proteins (MRPs), breast cancer resistance protein (BCRP), and bile salt export pump (BSEP) [82]. These transporters belong to the ATP-binding cassette (ABC) family, using the energy of hydrolyzing ATP to transport drugs and other endogenous substances.

#### 3.4.1. ABCC2

In the clinical application of sodium valproate, more than half of the patients show resistance to AEDs, not achieving the expected effects of AEDs in clinical treatment [83]. To date, among the studies on the resistance mechanism of sodium valproate, it is generally believed that reduced sensitivity of the target site and excessive transporters may be the main mechanisms causing the resistance [84]. As one of the members of the ABC family, the ATP-binding cassette-2 (ABCC2) shows high genetic polymorphism. The mutations in ABCC2 enhance its functions and in turn affect the drugs to enter the cytoplasm in brain tissue, reducing the plasma concentration of the drugs and ultimately affecting the therapeutic efficacy [85]. The *ABCC2* gene is located at position 24 of human chromosome 10 with a low content in normal brain tissue and mainly plays an important role in drug absorption and excretion [86].

Studies by Liu *et al*. based on *ABCC2 rs3740066* (*C3972T*) and *rs717620* (*C24T*) sites show that the mutation rates of *C3972T* and *C24T* sites in the selected population are 28.84% and 25.92%, respectively [87]. The *C3972T* genetic polymorphism is closely related to the efficacy and adverse reactions of neurological drugs. Although the mutation at *C3972T* site is synonymous, it still shows impact on the function of ABCC2, causing blocked drug absorption and excretion and affecting drug plasma concentration and therapeutic effects [88]. This study also shows that the standardized drug plasma concentration of patients with CC genotype at *C3972T* locus is higher than those of CT and TT genotypes, suggesting that mutations at *C3972T* locus affect the function of ABCC2, causing the changes in transporter<apos;>s function, ultimately leading to the blocked absorption and excretion of sodium valproate and reduced plasma concentration. Furthermore, this study demonstrates that the clinical efficacy of carriers of the T allele at *C3972T* locus is lower than that of non-carriers, indicating that the C→T mutation at this site may affect the therapeutic effect in patients. The *C24T* locus is located in the 5’ untranslated region of the *ABCC2* gene. Studies have shown that C24T polymorphism affects gene transcription activity, causing the individual variations in the function of ABCC2 [89]. These studies also show that the standardized plasma concentration and plasma concentration in patients of *C24T* CC genotype are higher than those of CT and TT genotypes, suggesting that the SNPs of C24T may reduce the transporter activity and the plasma concentration of sodium valproate.

Studies by Chen *et al*. on the genotypes of *rs2273697* (*G1249A*) and *rs3470066* (*C3972T*) sites of *ABCC2* gene show that the mutation rates of the *G1249A* and *C3972T* sites in the selected population are 9.37% and 24.16% [90], respectively, which are comparable to those reported by Liu *et al*. [87]. However, Chen *et al*. [90] have revealed no correlation between *ABCC2 C3972T* site and either the plasma concentration or efficacy of VPA. These inconsistencies may be due to either the lack of appropriate statistical analysis of the possible synergetic effects between the sites or the biased statistical results due to insufficient sample size. The *ABCC2 rs2273697* (*G1249A*) is a missense mutation with the conversion of the tyrosine to isoleucine at position 417. Chen *et al*. have reported that the plasma concentration of VPA in patients with genotype AA at *ABCC2 G1249A* site is significantly higher than those of carriers of genotypes GA and GG, probably due to the decreased efflux transport activity of ABCC2 [90].

In summary, patients with *ABCC2* mutation sites show lower VPA plasma concentration than those without mutation sites, and the drug efficacy in the carriers of *ABCC2* gene mutations may be affected. Therefore, in the clinical application of VPA, patients with *ABCC2* mutant genotypes could be treated with appropriately increased dosage to ensure the therapeutic effect.

#### 3.4.2. ABCB1

The ATP-binding cassette subfamily B member 1 gene (*ABCB1*), also known as the multi-drug resistance gene (*MDR1*), is expressed in human brain in the form of P-glycoprotein (P-gp), which is a transporter functioning as an ATP-dependent drug efflux pump used for AEDs such as sodium valproate [91]. These pumps reduce the level of therapeutic drugs in the cells by using the energy released by decomposing ATP to actively transport a large number of AEDs outside the brain capillary endothelial cells, ultimately developing drug resistance [92]. Human P-gp is encoded by the *ABCB1* (*MDR1*) and *ABCB4* (*MDR2*) genes, while the P-gp encoded by *MDR2* is not involved in drug delivery. The *ABCB1* genetic polymorphism affects the expression and/or function of P-gp, causing the substantial variations in the multi-drug resistance among individuals [93]. The *G2677A/T* mutation of the *ABCB1* gene causes amino acid changes related to the function of transporting P-gp and the changes of protein expression, while the *C3435T* and *C1236T* mutations located in the coding region are also related to changes in protein expression without altering the amino acids [94].

Results of the statistical analysis on the genotype of the *ABCB1* gene and the VPA plasma concentration by Ding *et al* [84]. show that the mutation rates of *C1236T, C3435T, G2677T*, and *G2677A* in the selected population are 55.24%, 35.31%, 47.20%, and 8.39%, respectively. Based on their efficacy, the patients of epilepsy in Chinese Han population treated with VPA monotherapy are grouped into (1) drug-resistance group (treated with regular AEDs either reaching the standardized plasma concentration or the maximum dosage commonly used but the frequency of seizures not reduced by 50%) and (2) the effective control group with the frequency of seizures reduced by more than 50%. Results of statistical analysis of the genotype distribution of each site of *ABCB1* in both groups show that there is a significant linkage disequilibrium among the genetic polymorphisms of *C1236T, G2677T*, and *C3435T* [95]. The VPA resistance group contains more haplotype CGC but less haplotype TTT than the effective control group, suggesting the association between haplotype CGC and the VPA resistance as well as the association between haplotype TTT and effective control of drugs. Furthermore, the incidence of CC type at *C3435T* locus in the drug-resistance group is higher than that of the effective control group, while the incidence of *C1236T* CT type is lower than that of the effective control group. No significant difference in the distribution of *G2677T/A* genotype is revealed between the two groups, suggesting that *C3435T* CC genotype may be more susceptible to drug resistance, while the application of VPA to patients of epilepsy with *C3435T* CT and *C1236T* CT genotypes may be more effective to control seizures than to patients with other genotypes.

## 4. Carbamazepine

### 4.1. Metabolic characteristics of carbamazepine

As a type of traditional clinical AED, carbamazepine (CBZ) shows optimal effect on psychomotor seizures. CBZ reaches a peak plasma concentration of 8–12 μg/mL in 4–5 hours after oral administration, while it takes 8–55 hours to reach a stabilized plasma concentration demonstrated by rapid distribution throughout the entire body with the plasma protein binding rate of ~76% and the bioavailability of 58–85%. By enhancing the inactivation efficiency of sodium channels, the CBZ restricts the divergence of high-frequency action potentials in the postsynaptic neurons, blocks the divergence of presynaptic sodium channels and action potentials, and blocks the release of neurotransmitters, ultimately regulating the nerve excitability and generating anti-convulsant and antiepileptic effects. Furthermore, CBZ has also been used in a wide range of clinical applications, including anti-peripheral neuralgia and anti-arrhythmia [70]. However, this medicine should be used with caution because adverse reactions are frequently revealed in digestive, hematopoietic, and endocrine systems [58,96]. Moreover, there are still problems such as large individual differences in plasma concentration and drug resistance in the current clinical application of CBZ. Therefore, it is important to develop individualized treatment plans based on the detection of CBZ plasma concentration in patients.

### 4.2. Effect of cytochrome P450 on the plasma concentration of carbamazepine

Studies have shown that CBZ metabolism is mainly catalyzed by CYP3A4, CYP3A5, CYP2C8, and CYP1A2, with both CYP3A4 and CYP3A5 playing the main roles [97,98]. CBZ is a highly efficient inducer of CYP3A4, CYP3A5, and other phase I and phase II metabolic enzymes in the liver, increasing the efficiency of substrate metabolism to reduce the plasma concentration of itself and other drugs metabolized by CYP3A4 and CYP3A5 [99].

#### 4.2.1. CYP3A4

CYP3A4 is the metabolic enzyme accounting for the highest content in the CYP450 family, participating in metabolism *in vivo* of 45–60% of currently available therapeutic drugs such as CBZ, cyclosporine, and lidocaine [100]. The activity of CYP3A4 is affected by age, gender, and diet of the patients, and particularly, genetic components as the dominant factor causing individual differences [101]. The *CYP3A4* gene is located on chromosome 7, with a length of ~27.2 kb, containing a total of 13 exons and 12 introns. To date, the CYP3A4*1 G (*rs2242480*) mutation changing from G to A at position 82,266 is located in intron 10 and identified as having relatively high mutation rate of 22.1–29.9% in Chinese population [74]. As another mutation site in intron 10 of *CYP3A4* gene, *rs4646440* is revealed to have a mutation rate of ~25% in Chinese population [102]. The *CYP3A4* mutations increase the activity of CYP3A4 enzyme and accelerate drug clearance. Results of the statistical analysis by Wang *et al*. [103] on loci *rs2242480* and *rs4646440* of *CYP3A4* reveal no correlation between the genotypes of these two sites and the CBZ plasma concentration. These results are consistent with those reported by Yun *et al*. [104], while Wang *et al*. [105] have report that the adjusted concentration and standardized concentration in patients of genotype GG at CYP3A4*1 G locus are significantly higher than those of genotypes AG and AA, though no significant difference is found between genotypes AG and AA. These results indicate that patients with wild type CYP3A4*1 G exhibit low metabolism, supporting that *CYP3A4* mutations can lead to increased enzymatic activity. However, these studies do not reveal the correlation between the *CYP3A4* genetic polymorphism and the efficacy of CBZ [103–105], probably due to the trivial changes in the plasma concentration of CBZ caused by the CYP3A4 polymorphism not making significant change in the drug efficacy [105]. Alternatively, after offsetting the effects of other genes, CYP3A4*1G is not sufficient to be a sole factor affecting the efficacy of CZB.

#### 4.2.2. CYP3A5

*CYP3A5* is located on human chromosome 7 q21.1–22.1 with a length of ~31.8 kb, containing a total of 13 exons and encoding 502 amino acids. The expression and activity of CYP3A5 vary considerably among individuals in different ethnic populations. High expression of CYP3A5 is revealed in ~10-20% of adults with *CYP3A5* accounting for more than 50% of the total CYP3A enzyme content in liver [106]. Jounaidi *et al*. have discovered the first SNP of *CYP3A5, i.e*., the substitution from cytosine (C) to adenine (A) at position 1280 of the 11th exon of *CYP3A5*, resulting in a mutation at position 398 with tryptophan changed to asparagine and the creation of a Tsp509 restriction site named CYP3A5*2 [107]. The *CYP3A5 A6986G* is a single-base mutation of intron 3 from adenine (A) to guanine (G) (CYP3A5*3), producing several splice variant mRNAs to shift the stop codon forward and resulting in the absence of *CYP3A5*. Studies have shown that the CYP3A5*3/*3 genotype (GG genotype) is not expressed to generate CYP3A5 [106], while the CYP3A5*3 is the most common type of SNP with a mutation rate of 71–76% in the Chinese population [108].

Ren *et al*. have reported that the mutation rate of the *CYP3A5 A6986G* locus in the selected population is 79.17% [109], which is comparable to those reported previously [68]. Ren *et al*. further recognize the genotypes AA and AG as the CYP3A5 expression group and genotype GG as the CYP3A5 non-expression group [109]. Results show that the dosage-corrected plasma concentration and the standardized plasma concentration of the CYP3A5 non-expression group are significantly increased by 27.9% and 32.2% in comparison to those of the CYP3A5 expression group, respectively. These results are similar to those reported by Park *et al*. [110], *i.e*., the plasma concentration of CBZ in the CYP3A5 non-expression group is 31% higher than that of the CYP3A5 expression group, which show a 29% higher oral clearance efficacy. These results are consistent with those reported by Liu *et al*. [111], indicating that patients with heterozygous genes have weakened metabolism, and their corrected drug plasma concentration is significantly higher than that of patients with wild type homozygous genes. Furthermore, no significant difference in efficacy and adverse reactions are identified between these two groups of patients with different genotypes of *CYP3A5*. However, using a population pharmacokinetic model to study the effect of *CYP3A5* genotype and CBZ clearance, Seo *et al*. [112] have reported conflicting results, *i.e*., the CYP3A5 non-expression group shows increased CBZ oral clearance efficacy by 8% than that of the CYP3A5 expression group. The inconsistency of these results may be due to either the use of combination of drugs in some patients, while the interaction between drugs cannot be completely ruled out, or the un-corrected variations in the metabolic rate caused by CBZ self-induction.

### 4.3. Effect of uridine diphosphate glucuronidase on plasma concentration of carbamazepine

In the UGT family, the UGT2B is encoded by a unique gene independent of the UGT1 family and located on chromosome 4q13 with *UGT2B4, UGT2B7, UGT2B15*, and *UGT2B28* showing the genetic polymorphisms [104]. Because UGT2B7 is the only member metabolizing CBZ in UGT family, we here review the studies focusing on the effects of UGT2B7 on the plasma concentration of CBZ.

As a member of the UGT2B family, the *UGT2B7* gene has a full length of 16 kb, containing 6 exons and encoding 529 amino acids. Studies have shown that both CBZ and its main effective metabolites (*i.e*., 10,11-epoxidized carbamazepine) are metabolized by UGT2B7. The *C802T* is the identification location of the coding region of *UGT2B7* gene and is confirmed to affect the stabilized plasma concentration of CBZ. Zhang *et al*. [113] have investigated *UGT2B7* using a statistical analysis and reported that the mutation rate of *C802T* site in the selected population is 29%, which is similar to those reported in Asian populations [114]. Results of Zhang *et al*. suggest that the standard drug plasma concentrations in the group of UGT2B7*2 genotypes are not statistically significant [113]. However, an increasing trend of the standardized drug plasma concentration is observed in the patients in the order of genotypes of mutant, heterozygote, and wild type. Qu *et al*. have report that UGT2B7*2 genotype can affect the stabilized plasma concentration of CBZ [115]. To date, the relationship between *UGT2B7* genotype and CBZ metabolism is not fully verified. The establishment of an improved statistical model with the exclusion of other factors involved is still needed in order to further investigate the relationships between *UGT2B7* genotype and CBZ metabolism.

### 4.4. Effect of transporters on the plasma concentration of carbamazepine

The drug transporters involved in the metabolism of CBZ are classified into two groups, including the families of ATP-binding cassette (ABC) transporters and solute transporters (solute carriers or SLCs). The ABC transporter family contains a total of ~50 members numbered starting with ABC, *e.g*., ABCB1 (MDR1), ABCC2 (MRP2), and ABCG2 (BCRP) [104].

#### 4.4.1. ABCB1

Multi-drug resistance protein 1 (MDR1) belongs to the P-gp family containing polymorphic members [91]. The P-gp is encoded by the *ABCB1* gene located on chromosome 7q21 and is a transmembrane P-glycoprotein (P170) with a molecular weight of 170 KD composed of 1,280 amino acid residues. The P-gp contains two homologous halves each containing 6 hydrophobic transmembrane domains and one ATP binding site [116]. The P-gp binds not only to drugs but also to ATP, which provides energy to pump intracellular drugs out of the cell, ultimately decreasing the drug concentration in the cell to make the cells resistant to drugs. Studies have shown that CBZ is a substrate of P-gp and the overexpression of P-gp may be one of the mechanisms regulating the resistance to AEDs [117].

Yun *et al*. [104] have selected three most common SNPs of *ABCB1* gene, *i.e., C1236T* (*rs1128503), G2677T/A* (*rs2032582*), and *C3435T* (*rs1045642*), to investigate the relationships between the genetic polymorphisms of these three loci and the plasma concentrations of CBZ as well as its two metabolites (*i.e*., CBZE and CBZD). The results demonstrate that patients with the *ABCB1* 3435CT mutation genotype and the 3435 TT homozygous mutation genotype show lower plasma concentrations than that in patients with the 3435CC wild genotype. However, other studies show that the *C3435T* genetic polymorphism is not correlated with the CBZ plasma concentration [118,119]. These conflicting experimental results may be caused by: (1) the activity of P-gp and the distribution of ABCB1 polymorphisms are different in different populations, (2) other transporters such as ABCC2 also play important roles during the CBZ transport process, (3) CBZ may induce the expression of P-gp in both the intestine or brain tissues, thereby reducing drug absorption, while the expression level of P-gp may also be affected by the long-term use of CBZ [120], and (4) the stage of treatment or the time to collect blood samples vary in different studies. Furthermore, studies have reported that patients carrying *ABCB1* 3435CC wild type show higher CBZE and CBZD plasma concentrations than that of patients with 3435CT heterozygous mutations, suggesting that CBZE may be a substrate of P-gp [121].

#### 4.4.2. ABCC2

The drug transporter ABCC2 (MRP2) is encoded by the *ABCC2* gene and is overexpressed in the endothelial cells of the blood-brain barrier, liver, intestine, kidney, placenta, and lung. However, the expression level of ABCC2 is relatively low in normal brain tissue, whereas the expression level of ABCC2 in the brain tissue of patients with epilepsy is relatively increased [122]. In Korean populations, the 1249 G > A mutation decreases the ability of ABCC2 to efflux and increase the intracellular concentration of CBZ, causing severe side effects [123]. Several possible reasons could cause these varied experimental results. First, it is uncertain whether CBZ is a substrate of ABCC2. Second, these studies may have used different standards to determine the controlled epilepsy, subsequently affecting the interpretation of clinical data [124]. Third, there are potentially genetic differences between different ethnic groups of populations. Lastly, other polymorphic sites or epigenetic factors of *ABCC2* may affect the metabolism and efficacy of CBZ.

## 5. Phenytoin

### 5.1. Metabolic characteristics of phenytoin

Phenytoin (PHT), also known as dalantine, is a type of sodium salt of diphenylhydantoin and is the first-line drug for the treatment of severe seizures of epilepsy. In addition to its antiepileptic and anti-arrhythmic properties, PHT has shown wide clinical applications. The novel pharmacological effects of PHT have been discovered in recent years, such as treating acute cerebral infarction and atherosclerosis, burns, and hiccups, promoting wound healing, achieving satisfying therapeutic effects, and showing broad application prospects [125]. However, due to its saturated metabolism and non-linear dynamics characteristics as well as the narrow treatment window and large individual variations, the application of PHT is likely to cause many types of adverse reactions in the central nervous system and hematopoietic system [126,127].

PHT is highly lipid-soluble and is transported to multiple tissues and organs of the body after joining plasma proteins. It can quickly pass through the blood-brain barrier [128], inhibit the passive influx of Na^+^ outside the cell to stabilize the cell membrane and increase its excitement threshold, and prevent the spread of abnormal brain potential activities to surrounding normal tissues, thereby inhibiting seizures of epilepsy [129]. PHT is eliminated through liver metabolism, while the metabolites show no drug activity and are mainly excreted in urine with a small amount (<5%) excreted in its original form. The effective therapeutic functions of PHT require a certain degree of saturation of liver metabolic enzymes (*i.e*., hydroxylase). PHT exhibits the first-order kinetics at low dosage but follows the zero-order kinetics once the enzyme becomes saturated. Within the treatment range, the ability of liver to metabolize PHT reaches the highest level with the increase of drug plasma concentration, while further increase of a trivial dosage may cause an enormous change in the concentration of PHT [130].

### 5.2. Effect of cytochrome P450 on the metabolism of PHT

In general, the phenotypes of CYP450 enzymes are categorized into four types, *i.e*., poor metabolizers (PMs), intermediate metabolizers (IMs), extensive metabolizers (EMs), and ultrarapid metabolizers (UMs). The effects of genetic polymorphism of *CYP450* on drug metabolism, efficacy, and toxicity have been extensively studied. For example, studies have shown that under the same conditions, poor drug metabolizers often carry related mutant genes, causing the drug to accumulate in their bodies, showing either over-estimated efficacy or prone to drug poisoning with the regular dosage, while the fast metabolizers often suffer the excessive drug resistance showing low efficacy [131]. We here review the studies on the effects of *CYP2C9* and *CYP2C19* on the plasma concentration of PHT in the treatment of epilepsy.

Studies have shown that among the ten known alleles of *CYP2C9*, besides the wild-type CYP2C9*1, other two most common alleles (i.e., CYP2C9*2 and CYP2C9*3) encode enzymes with reduced activities of 70% and 3–5%, respectively, in comparison to that of the wild type [132]. The carriers of the variant genes account for 40% among Caucasians and only 5% in Asians with the CYP2C9*3 as the most frequent mutation in Asian populations [132]. The studies of enzymatic kinetics *in vitro* have shown that the intrinsic clearance rate of PHT metabolism by CYP2C9*3 decreases by 6–13.5%, in comparison to that of *CYP2C9* wild-type (i.e., CYP2C9*1) [133]. Clinical studies have also confirmed that CYP2C9*3 has shown the ability to significantly affect the clearance of PHT drugs [134]. Among the two most common mutation sites (i.e., CYP2C9*2 and CYP2C9*3), the mutations in the active site resulting in a decrease in affinity with the substrate is considered as the mechanism causing the decrease in enzymatic activity by CYP2C9*3. Furthermore, the activity of CYPs may be affected by the electrons delivered by their coenzymes, *i.e*., the CYP450 oxidoreductase (POR). The CYP2C9*2 is a mutation encoding the outer surface proteins. The mechanism of CYP2C9*2 causing a decrease in the metabolic rate of CYP2C9 substrate may be associated with the decrease in POR binding ability [135]. Studies by Ramasamy *et al*. [136] show that the metabolism of PHT in heterozygous carriers of *CYP2C9* gene (*1/*3) is significantly lower than that of wild-type homozygous carriers (*1/*1). The activity of the enzymes encoded by CYP2C9*3 is decreased to only 3–5% of that of the wild type. With the same dose, the plasma concentration of slow metabolizers is 34% higher than that of fast metabolizers [137].

Besides the wild-type CYP2C19*1, the CYP2C19*2 and CYP2C19*3 are two most common alleles among the eight alleles of *CYP2C19*. In the Asian populations, 55.4% of Japanese patients of epilepsy carry the *CYP2C19* variant with 13–23% poor metabolites in Asian populations and 2%-5% in white and black populations [138,139]. Although the CYP2C19 is only responsible for 10% of PHT metabolic clearance, studies *in vivo* have demonstrated that patients with CYP2C19*2 or CYP2C19*3 show significantly reduced ability to metabolize PHT [140]. Furthermore, the differences in drug metabolism caused by genetic polymorphisms increase the steady-state plasma concentration of PHT to toxic level and cause toxic reactions [141].

Results of statistical analysis on both *CYP2C9* and *CYP2C19* by Ren *et al*. [142] show that the allele frequencies of CYP2C9*3, CYP2C19*2, and CYP2C19*3 are 7.30%, 33.50%, and 3.70%, respectively, in accordance with the distributions of these genetic sites in Chinese population [143]. Based on the genetic polymorphisms of *CYP2C9* and *CYP2C19*, patients are categorized into three groups, including the extensive metabolizers (EMs) containing the genotype CYP2C9*1/*1 combined with CYP2C19*1/*1, the intermediate metabolizers (IMs) with either the CYP2C9*1/*3, CYP2C19*1/*2, or CYP2C19*1/*3, and the poor metabolizers (PMs) containing either the CYP2C19*2/*2 or CYP2C19*1/*2 combined with CTP2C9*1/*3. The study of Ren *et al*. [142] has demonstrated that the drug plasma concentrations of the extensive, intermediate, and poor metabolizer groups significantly increase sequentially, while the significant differences are revealed in patients not reaching the minimum effective concentration and those exceeding the minimum toxic concentration among the three groups of patients. Specifically, the drug plasma concentration in 95.5% of patients of extensive metabolizers is less than the minimum effective concentration, while as high as 23.1% of poor metabolizers reaching the minimum toxic concentration. Zhou *et al*. [144] have conducted a statistical analysis on the *CYP2C19* gene of children with epilepsy treated with PHT monotherapy. The patients are categorized into three groups based on the CYP2C19 genotypes detected, including the extensive metabolizers (EMs) carrying the wild homozygous genotype *1/*1, the intermediate metabolizers (IMs) carrying the point mutation genotypes of *1/*2 and *1/*3, and the poor metabolizers (PMs) with the double site mutation genotypes of *2/*2, *2/*3, and *3/*3. After each group of patients are given the same initial dose of PHT (5 mg/kg taken in 2–3 times), then different maintenance doses of PHT are administrated to different groups, *i.e*., the group of EMs are given 250 mg/m^2^ (taken in 2–3 times), while the groups of IMs and PMs are given sequentially reduced doses. After the patients have taken PHT for more than 5 half-lives and the PHT in the body reaches the steady-state plasma concentration [145], the plasma concentration of PHT is evaluated. Results show that there is no statistically significant difference in either the steady-state plasma concentration of different metabolizers or the incidence of adverse reactions.

In summary, these results indicate that the examination of drug metabolism-related genes prior to the treatment of patients with epilepsy as well as the establishment of individualized dosage plan formulated based on the results of the genotype examination would greatly facilitate the accurate control of the drug plasma concentration within the treatment window and obtaining improved therapeutic effects and decreased adverse reactions in patients.

### 5.3. Effect of transporters on the metabolism of PHT

To date, it is commonly accepted that P-gp is associated with the formation of refractory epilepsy. This is because P-gp is not only the expression product of *ABCB1* gene, but also part of the drug efflux transport system. By hydrolyzing ATP, the drug is pumped out of the cell, thereby the effective drug plasma concentration is not reached in the cells of affected tissues, ultimately affecting the therapeutic effect of the drug. Studies have shown that P-gp is closely related to refractory epilepsy by affecting the transport of a variety of AEDs [146]. We here review the studies on the effects of ABCB1 on the plasma concentration of PHT.

Kimchi *et al*. [147] have studied the SNPs of the *ABCB1* gene and reported that the *C3435T* site affects DNA folding by changing codon selection and application, leading to changes in protein structure. Three SNP sites (*i.e*., 3435C>T, 1236C>T, and 2677 G > T) on *ABCB1* gene subtly alter the structure of P-gp by affecting the speed and rhythm of translation of the codon substitution, thereby changing the affinity of either regulatory factors or substrates. Siddiqui *et al*. [95] have investigated the relationship between the *ABCB1* genetic polymorphism and AED resistance based on studies of 315 patients of epilepsy. Results show that the proportion of the CC genotype of *ABCB1 C3435T* in AED resistant patients is significantly higher (27.5%) than that of drug sensitive patients (15.7%), while the proportion of TT genotype is significantly lower (19.5%) than that of drug sensitive patients (29.6%). Furthermore, Zimprich *et al*. have reported that the haplotype composed of *C1236T, C3435T*, and *G2677T/A* is related to drug resistance in patients of epilepsy [148], while Hung *et al*. have demonstrated that the genetic polymorphisms of *ABCB1* genes *G2677T* and *C3435T* are related to AED resistance [149]. However, several studies have revealed no association between the genetic polymorphism of these genes and the AED resistance [150,151]. For example, Jiang *et al*. have conducted statistical analyses on the *C1236T, C3435T*, and *G2677T/A* sites of the *ABCB1* gene and revealed no statistical difference in the plasma concentration of PHT among the patients of the three SNP genotypes [152]. Based on the criteria for defining epilepsy resistance proposed by Shahwan *et al*. [153], who categorize the enrolled patients into either resistance group or control group and report that different alleles at the *C1236T* locus show statistically different distribution frequencies in the resistance and control groups. Specifically, the distribution frequency of the C allele in the resistance group (37.9%) is significantly higher than that in the control group (23.3%), while the distribution frequency of the T allele in the control group (76.6%) is significantly higher than that in the resistance group (62.0%). At the *G2677T/A* locus, the distribution frequency of the G allele in the resistant group (55.0%) is significantly higher than that in the control group (32.0%), while the distribution frequency of the T allele in the control group (53.8%) is significantly higher than that in the resistant group (29.6%). The distribution frequencies of *C3435T* alleles are not statistically significant. It is noted that studies on the *ABCB1* genetic polymorphism, epilepsy, and epilepsy resistance have reached inconsistent results. Some studies support the hypothesis of the association between genetic factors (*i.e*., allele, genotype, or haplotype) and epilepsy as well as epilepsy resistance [154,155]. However, studies on patients of epilepsy in different regions and ethnic groups do not support the association hypothesis [155,156]. Studies with more statistical methods to conduct the meta-analyses have verified these inconsistent and contradictory conclusions, revealing no association between *ABCB1* polymorphism and epilepsy as well as drug-resistant epilepsy [156–158].

Currently, the relationship between the *ABCB1* genetic polymorphism and PHT plasma concentration as well as the epilepsy resistance remains unknown. Further studies are necessary based on an improved model established to adjust factors such as geographic location, ethnic difference, and site interactions.

## 6. Pharmacokinetic characteristics of new antiepileptic drugs

In the past 10 years, new AEDs (*e.g*., OXC, LEV, and LTG) have been developed and widely used in clinical treatments. These new AEDs mostly show linear pharmacokinetic characteristics with the plasma concentration proportional to the dose as well as sound oral absorption and quickly reaching peak plasma concentration. Furthermore, due to their high bioavailability, the absorption of these AEDs are not affected by the type of food taken, while the plasma protein binding rate is low and the drug is generally widely distributed in the tissues [159,160].

The new AEDs are partially metabolized in the liver and are excreted from the urine through the kidneys either in the original form or as metabolites [159–161]. Compared with the traditional AEDs, the new AEDs rarely interact with other drugs [162]. To date, the new AEDs globally investigated and significantly affected by metabolic enzymes and their genetic polymorphisms include LTG, OXC, and LEV. We here review the studies on the relationship between the plasma concentration of these new AEDs and the genetic polymorphism of genes encoding enzymes involved in the metabolism of these new AEDs.

## 7. Lamotrigine

Lamotrigine (LTG) is one of the most commonly used new generation AEDs with a broad therapeutic spectrum. It is effective for some common types of seizures, such as simple partial seizures, complex partial seizures, partial seizures with secondary generalization, primarily generalized tonic-clonic seizures, and absence seizures. LTG is also used as a mood stabilizer for the treatment of bipolar disorder, particularly effective for patients in depressive phase of bipolar disorder without inducing mania [163]. LTG mainly shows antiepileptic effects by blocking the voltage-sensitive sodium ion channels of the presynaptic membrane, thereby inhibiting the release of excitatory amino acids (*i.e*., glutamate and aspartate). Recent studies have suggested that LTG can also exert antiepileptic activity by blocking the voltage-sensitive calcium channels [164]. LTG can be rapidly and completely absorbed by oral administration without evident first pass elimination. Its bioavailability is high (98%) with the plasma protein binding rate of 55%.

### 7.1. Metabolic characteristics of lamotrigine

LTG is mainly metabolized by UGT enzymes in the human body to convert into conjugated products of hydrophilic glucuronic acid and excreted by the kidneys, while the phase I metabolic enzyme CYP does not participate in the metabolism of LTG *in vivo*. LTG shows no evident hepatic enzyme induction, while its self-induction appears in the early stage of treatment with no distinct clinical significance [77]. About 75–90% of the dose of LTG is excreted in the form of glucuronic acid conjugated products, consisting of mainly 2-N-glucuronide and 5-N-glucuronide accounting for ~10%, while less than 10% of the dose of LTG is excreted in its original form with the fecal excretion accounted for only 2%. The highly safe LTG shows a half-life of 15–30 hours and is the first choice of AED for women of childbearing ages.

Similar to other AEDs, LTG passes through the blood-brain barrier to reach the epileptic center to exert its antiepileptic activity. Studies have shown that the concentration of LTG in the cerebrospinal fluid is correlated with the concentration of LTG in the circulating blood [165]. Therefore, the concentration of LTG in plasma is a strong indication of the efficacy of LTG. Some patients with epilepsy take large doses of medicine without achieving satisfactory therapeutic effects, while other patients show severe adverse reactions with small doses. Therefore, it is clinically important to monitor the plasma concentration of LTG in order to achieve satisfactory therapeutic effects and to avoid severe adverse reactions in patients of epilepsy.

### 7.2. Effect of uridine diphosphate glucuronidase on the metabolism of lamotrigine

The enzymes UGT1 and UGT2 play important roles in the metabolism of endogenous and exogenous substances in the human body. These enzymes use UDP-glucuronic acid groups as the glycosyl donors showing high activity in catalyzing the phase II binding reactions. The compounds glycosylated by UGT1 and UGT2 enzymes show increased hydrophilicity and are easily excreted from the human body. The function of UGT3 enzyme is currently unclear. It has been reported that UGT8A1 enzyme uses UDP-galactosyl as a glycosyl donor to catalyze the synthesis of cell membrane components, while enzyme UGT1A4 is the main isoenzyme involved in the acidification reaction of LTG [166]. Studies have shown that enzymes UGT2B7 [167], UGT1A3 [168], and UGT1A1 [169] participate in the acidification reaction of LTG with both UGT2B7 and UGT1A3 showing lower catalytic activities, while UGT1A4 showing 10 times higher catalytic activities than that of UGT1A3 [167,168].

#### 7.2.1. UGT1A4

Enzyme UGT1A4 is mainly expressed in the human liver, bile duct, small intestine, and colon, mainly involved in the metabolism of drugs containing primary and secondary amine structures. It has been reported that the mutation frequencies of *UGT1A4 C457T, G419A, G163A*, and *C471T* in Chinese population are 0.190, 0.191, 0.194, and 0.998, respectively [170]. Ehmer *et al*. have experimentally confirmed for the first time the association between *UGT1A4* genetic polymorphism and the enzymatic functions [171]. Specifically, it is revealed that the mutation of exon 1 of *UGT1A4* gene affects the function of the enzyme. Two mutation sites are identified with one located at the 70th base causing the change from proline to threonine at position 24 (P24T), and the other at the 142nd base causing the change from leucine to valine at position 48 (L48V). These two mutations also affect the enzymatic activity of UGT, *i.e., UGT1A4* gene mutations P24T and L48V cause the decrease of the UGT enzymatic activity [171].

Yang *et al*. have investigated the relationship between *UGT1A4 T142G* locus and the plasma concentration of LTG to show that the distribution frequencies of *UGT1A4* 142 T > G alleles T and G are 87.74% and 12.26%, respectively [172], which are similar to that of the Japanese population (12.9–16.5% for G allele) as previously reported [173,174]. Furthermore, the standardized plasma concentration of LTG in patients with TT genotype at *UGT1A4 T142G* locus is significantly higher than that of patients with TG and GG genotypes, and the clinical efficacy of patients with TT genotype is significantly higher than that of patients with TG and GG genotypes. Gulcebi *et al*. have reported the similar results, *i.e., UGT1A4 T142G* site mutation increases the catalytic activity of UGT enzyme, resulting in a decreased plasma concentration of LTG, *e.g*., the plasma concentration of LTG in *T142G* mutation carriers is 52% lower than that of wild-type patients [175]. These results are consistent with those reported by He *et al*. [176], further revealing that in the combined medication, the plasma concentration of VPA is related to the plasma concentration of LTG, *i.e*., VPA increases the plasma concentration of LTG by 1.0%. However, the results of Liu *et al*. [177] have revealed no association between the *UGT1A4 T142G* polymorphism and the LTG plasma concentration with the LTG used alone, whereas when used in combination with VPA, the LTG plasma concentration of mutant patients is higher than that of wild type, similar to the results of the aforementioned studies. These results indicate that patients with *UGT1A4 T142G* allele G show accelerated metabolism of LTG and reduced plasma concentration in the body. Therefore, it is recommended that these patients be treated with appropriately increased dosage of LTG under the clinical treatment, whereas the wild-type (TT) patients with relatively weak metabolic capacity should be treated with appropriately reduced dosage.

#### 7.2.2. UGT2B7

The subtype enzymes of UGT287 are also involved in the LTG metabolism. To date, studies have explored the effects of genetic polymorphism of *UGT287-C161T* and *UGT287 G211T/A* on LTG plasma concentration in the populations of epilepsy in Caucasian, Japan, Thailand, and China [178–181], confirming that the SNPs are associated with LTG concentration in Caucasian, Japanese, and Thai populations but not in Chinese population of epilepsy. Therefore, it is concluded that the effects of SNPs on drug plasma concentration vary among different ethnic populations, ultimately causing different drug efficacy in different populations and individuals. Milosheka *et al*. have conducted a population pharmacokinetic analysis based on 100 patients treated with LTG [182]. Results show that the *UGT2B7 C161T* and *A372G* genotypes are associated with the clearance rate of LTG, which is significantly lower in carriers of −161 TT than that of carriers of −161CC, and significantly higher in carriers of −372 GG than that of −372AA carriers. However, studies by Zhou *et al*. and Liu *et al*. on Chinese patients of epilepsy have not revealed the significant effect on LTG plasma concentration by *UGT2B7 C161T, G211T, A372G, A735G*, and *C802T* SNPs [177,182,183]. These results indicate that the sites of the *UGT2B7* gene have not shown any significant effects on the metabolism of LTG in the Chinese population. Further studies are still needed to verify these conclusions.

### 7.3. Effect of transporters on the metabolism of lamotrigine

Previous studies have shown that genetic polymorphisms of drug-metabolizing transporters, such as the P-gp encoded by the *MDR1/ABCB1* gene, the BCRP encoded by *ABCG2* gene, and the multi-drug resistance-related protein-2 encoded by *MDR2/ABCC2* genes, significantly affect the pharmacokinetics and bioavailability of AEDs. The overexpression of these transporters has been shown to affect the plasma concentration of LTG and cause different therapeutic effects [184]. As a subfamily of influx transporters, organic cation transporters (OCTs) play important roles in the distribution and excretion of cationic drugs as well as non-charged compounds.

#### 7.3.1. OCT1

The organic cation transporters (OCTs) are the subtypes of internal transporter proteins and are mainly involved in the transport process of organic cation proteins and uncharged complexes [185–187]. Studies by Dickens *et al*. show that OCT1-mediated active transport plays an important role in maintaining the intracellular concentration of LTG, suggesting that LTG may serve as a substrate of OCT1 in cerebral vascular endothelial cells [188]. Lu *et al*. have reported that the mutation rate of *OCT1 A1222G* (*rs628031*) site in the selected population is 67.65% [189], which is similar to that reported previously [190], showing a correlation between the *A1222G* locus genotype and the LTG plasma concentration but not associated with the efficacy of LTG. Furthermore, the plasma concentration in patients of GG genotype is 25.6% lower than those of AA and AG genotypes, suggesting that patients with GG genotype require a higher maintenance dose of LTG in order to achieve the recommended effective plasma concentration. However, no significant correlation is identified between the *OCT1 C1022T* site and the LTG plasma concentration as well as its efficacy. To date, studies on the effects of *OCT* gene on the plasma concentration of LTG are sparse [188–190]. The explicit relationship between *OCT* and the plasma concentration and metabolism of LTG remains to be investigated by further studies.

#### 7.3.2. ABCG2

The BCRP belongs to the ABC transporter superfamily and is encoded by the *ABCG2* gene, distributed in a variety of tumor cells and normal human tissues, such as the brain, liver, and small intestine, playing important roles in transport and efficacy of various types of drugs, including LTG [191]. Studies based on the *ABCG2* knockout mice have shown that *ABCG2* may be involved in the distribution of LTG in the brain [192], while the genetic polymorphism of *ABCG2* may affect the expression and function of ABCG2 protein, thereby affecting the pharmacokinetics of the drugs [193].

Lu *et al*. have reported that the patients with *ABCG2 C421A* (*rs2231142*) AA show a higher plasma concentration of LTG compared with those of CC and CA genotypes [189]. However, no evident association is revealed between *ABCG2 G34A* (*rs2231137*) genetic polymorphism and plasma concentration of LTG. Zhou *et al*. have also reported that the *ABCG2 rs2231142* genetic polymorphism show a significant effect on LTG plasma concentration [136]. Among the genotype distribution, AA, CA, and CC genotypes account for 10.7%, 44.3%, and 45%, respectively [190]. Similar to the above results, patients with *ABCG2 rs2231142* allele A may be treated with a relatively lower dosage of LTG in order to reach the maintenance plasma concentration required for the treatment. Studies have shown that the expression of allele A increases the risk of transporters being degraded by the proteasome, ultimately reducing the binding of transporters to cell membranes [194]. It is noted that He *et al*. have reported that there is no significant correlation between the *ABCG2 rs17731538* locus and the LTG plasma concentration and drug resistance in Chinese patients of epilepsy [195].

#### 7.3.3. ABCB1

The P-gp is involved in the transport of a variety of drugs, while the *ABCB1* genetic polymorphism causes varied effects and concentrations of multiple drugs in different genotypes. Studies have shown that LTG is a substrate of P-gp, which participates in the transport of LTG through the blood-brain barrier and endogenous cells [196], while the P-gp actively transports drugs out of the cell using the energy released from ATP hydrolysis, reducing the intracellular drug concentration. Studies have shown that the mutation of *ABCB1 C1236T* is associated with both the dose-corrected concentration of LTG and the maintenance dose of LTG [197,198]. To date, the most comprehensively studied *ABCB1* gene sites are *C1236T, G2677T/A*, and *C3435T* with their genetic polymorphisms affecting the expression and functions of P-gp. The *G2677T/A* is a missense mutation encoding either serine or threonine mutated from alanine [199,200].

Lovrić *et al*. have investigated the correlation between the LTG plasma concentration and *ABCB1* SNPs based on 222 Croatian patients [198]. Results show that the LTG dose-corrected concentration of 1,236 CC gene carriers is significantly higher than those of CT and TT carriers. However, no significant association is revealed between the *ABCB1 G2677T* and *C3435T* genes and the LTG plasma concentration [198]. Furthermore, Huang *et al*. have reported that when the *ABCB1 2677 G* (*rs2032582*) TT genotype and the *C3435T* (*rs1045642*) TT genotype coexist, the distribution volume of LTG in the body increases by 136.0%, while the plasma concentration may decrease [201]. However, other studies based on Chinese population have not found the correlation between the *ABCB1* genes and the LTG plasma concentration [189,195,202].

In summary, there is no specific mutation site affecting the LTG plasma concentration by LTG metabolic enzyme and transporter genetic polymorphisms, probably due to the complexity of the LTG metabolism and transport process not affected by a single metabolic enzyme and transporter. The LTG plasma concentration is generally affected by many non-genetic factors such as age, drug combination, and estrogen level of the patients. Therefore, in order to explicitly clarify the effects of metabolic enzymes and transporter SNPs on the LTG plasma concentration, it is necessary to further pursue clinical studies with multiple centers and large sample size to correct the effects of age, combination of medication, and estrogen levels of the patients, to introduce the population pharmacokinetics to quantitatively describe the effect of genetic polymorphism on LTG metabolism, and ultimately to provide evidence for establishing individualized clinical administration of LTG.

## 8. Oxcarbazepine

As a type of new generation AED, the oxcarbazepine (OXC) was approved by the U.S. Food and Drug Administration in 2000 for the treatment of epilepsy [203]. As the first-line drug choice for patients with newly diagnosed or untreated focal epilepsy, OXC could be used either alone or in supplementary treatment [204,205]. OXC is a 10-keto derivative of CBZ, while the pharmacokinetics of OXC and CBZ are different with the tolerability of OXC significantly higher than that of CBZ [206].

In the actual clinical applications for the treatment of the same type of epileptic seizure, the dosage of OXC used by different individuals varies greatly. One of the reasons causing these variations is probably the gene mutation with one of its genetic basis being the genetic polymorphism of the genes encoding related enzymes and receptors, ultimately causing the individual differences in the optimal dose of OXC in different individuals with epilepsy [207,208]. Furthermore, the effective plasma concentration of OXC fluctuates in the relatively narrow range of 5–30 μg/ml. The understanding of pharmacogenomics of OXC will facilitate the clinical drug selection, drug dosage adjustment, and reduction of adverse drug reactions.

### 8.1. Metabolic characteristics of oxcarbazepine

As a derivative of CBZ, the OXC shares the similar chemical structure with CBZ and the pharmacological mechanism of blocking the voltage-sensitive sodium ion channels [209]. As an inactive prodrug, OXC is quickly reduced by cytoplasmic enzymes in the liver to a pharmacologically active monohydroxy oxide, *i.e*., the 10,11-dihydro-10-hydroxy-carbamazepine (MHD) [210]. The MHD contains two types of enantiomers, *i.e*., S-type and R-type, with the ratio of ~4:1 in the body. Animal experiments have shown that the antiepileptic activities of both S-type and R-type are the same but with inconsistent pharmacokinetics. Previous studies have shown that UGT2B15 is the metabolizing enzyme for S-type glucuronidation, while UGT2B7 and UGT1A9 are the metabolizing enzymes for the R-type glucuronidation [211]. Studies have shown that R-type glucuronidation is not affected by patient<apos;>s genotype and gender, whereas the gender and *UGT2B15 D85Y* genotype are the main factors affecting the S-type glucuronidation [212]. The metabolism of OXC generating its active products is non-enzyme-inducible, which is hardly affected by CYP450 metabolic enzymes, though with trivial enzymatic induction or inhibitory effects, inducing the activity of CYP3A4/5 [213].

### 8.2. Effect of cytochrome P450 on the metabolism of oxcarbazepine

The effects of CYP450 enzymes on the metabolism of OXC have been determined using hepatocytes and HepaRG cells *in vitro*. Results show that as three main enzymes involved in the metabolism of OXC, CYP1A2, CYP2B6, and CYP3A4 are induced by OXC [214]. Experiments *in vitro* have demonstrated that OXC can induce CYP1A2, CYP2B6, and CYP3A4, while 10-hydroxyoxcarbazepine induces CYP2B6 and CYP3A4 but not CYP1A2, as measured at mRNA level in the hepatocytes and HepaRG cells cultured for 72 hours [215].

#### 8.2.1. CYP3A4 and CYP3A5

CYP3A is an important subfamily of CYP450 and is the most abundant hepatic drug-metabolizing enzyme in the liver. As two important members of the CYP3A family, CYP3A4 and CYP3A5 share high homology and are abundant in the liver with ~150 drugs as their substrates, accounting for about 50% of all drugs. The activity of CYP3A4 is mainly affected by genetic factors. Studies of the effects of the *CYP3A4* genetic polymorphism on its enzymatic activity have shown that CYP3A4*1G mutation increases enzymatic activity and accelerates drug metabolism [105,216]. Studies by Wang *et al*. have not revealed the positive correlation between the plasma concentration of OXC and the *CYP3A4/3A5* genetic polymorphisms [217], indicating that OXC can inhibit CYP3A4/3A5 enzymes, but in turn affecting the metabolism of other drugs, while OXC itself is not affected by these enzymes.

#### 8.2.2. CYP2C19

Although the OXC is not metabolized by CYP450, previous studies have shown that both OXC and its metabolite MHD inhibit the CYP2C19 enzyme, indicating that it is possible for the OXC to bind with enzyme to affect its own metabolism [218]. To date, the main *CYP2C19* variants in the Chinese population are CYP2C19*2 and CYP2C19*3, while no significant difference is revealed in the inhibitory effects of AEDs on patients with CYP2C19*2 and CYP2C19*3 genotypes [209]. A large number of studies have shown that there are three phenotypes of CYP2C19 enzyme, including the fast metabolizing type (636 GG), the medium metabolizing type (636 GA), and the slow metabolizing type (636AA) [218]. Zhou *et al*. have investigated the relationships between the CUP2C19*3 locus genotype and the OXC plasma concentration [210]. Results show that the frequencies of alleles G and A are 68% and 32%, respectively. Patients of different metabolizing types show significantly different effects on MHD plasma concentration. Specifically, the drug plasma concentrations of patients with slow and fast metabolizations are the highest and the lowest, respectively, indicating that the slow metabolization causes a decrease in CYP2C19 enzymatic activity, thereby increasing the plasma concentration of MHD. These results further suggest that the CYP2C19*3 genetic polymorphisms of different patients lead to significant differences in MHD plasma concentrations, causing reduced therapeutic effects or aggravated adverse reactions. Therefore, these problems should be monitored cautiously in clinical settings.

Zhou *et al*. have studied the relationships between the *CYP2C19* genotype and the metabolism of OXC in children with epilepsy in Chinese population [219]. Results show that the genotype frequencies of CYP2C19*2 locus *1/*1, *1/*2, and *2/*2 are 53.85%, 33.85%, and 12.31%, respectively, while the allele frequency of *2 is 29.23%, which is in accordance with the distribution of this gene locus in the Chinese population reported previously [220]. Results of univariate analysis have demonstrated that the CYP2C19*2 locus genotypes show no significant effect on the MHD plasma concentration. However, as the number of mutant alleles increases in patients, the average drug plasma concentration shows an increasing trend. These results indicate that OXC is not metabolized by CYP450 enzymes. Therefore, the difference in enzymatic activity caused by *CYP450* genetic polymorphism does not necessarily lead to the differences in the plasma concentration of OXC. Instead, as the metabolite of OXC, the MHD may be joined with CYP2C19 to affect its own metabolism, leading to the differences in therapeutic efficacy and possible risk of adverse reactions [221]. Therefore, it is recommended that OXC should be prescribed cautiously in clinical treatment of patients with epilepsy.

### 8.3. Effect of uridine diphosphate glucuronidase on oxcarbazepine metabolism

OXC is rapidly and completely absorbed orally and is then quickly metabolized into the active metabolite MHD in the body by the aromatic ketone degrading enzymes. MHD is mainly metabolized by UGT to produce glucuronide and excreted through the kidneys. Studies have reported that the MHD plasma concentration in carriers of *UGT1A9* or *UGT2B7* mutant genes is significantly higher than that of non-mutant patients [179,222]. However, more comprehensive studies on the effects of UTG on the metabolism of OXC are currently lacking. Further investigations are necessary to clarify the effects of UTG on the metabolism of OXC.

#### 8.3.1. UGT1A9

UGT1A9 is expressed widely in liver, kidney, stomach, small intestine, colon, testis, prostate, and ovary. Results of *in vitro* studies based on human liver microsomes show that the *UGT1A9* intron polymorphic variant *CI399T* is associated with enhanced glucuronidation of substrates of UGT1A1 and UGT1A9 [223]. Zhang *et al*. have explored the relationship between *UGT1A9* genotype and OXC plasma concentration in Chinese patients of epilepsy [224]. Results show that the mutation rate of *UGT1A9 CI399T* gene in selected children with epilepsy is 56.9%, which is higher than 43.5% reported by Lu *et al*. [225] but similar to 55% reported by Guo *et al*. [226]. No correlation is identified between the *UGT1A9 CI399T* genetic polymorphism and either the MHD plasma concentration or the clinical efficacy in children with epilepsy, probably due to the weakened expression of *UGT* gene in children [227]. Lu *et al*. have categorized patients of epilepsy into seizure group and seizure-free group based on SNPs and therapeutic efficacy [225]. Results show that there is a significant difference between the seizure group and the seizure-free group at the *UGT1A9 CI399T* site. In the seizure-free group, there are more CC genotypes than either CT or TT genotypes, while there is no statistical difference between CT and TT genotypes, suggesting that the mutant T allele is associated with the increase of UGT1A9 activity. These results are consistent with those reported previously [223].

#### 8.3.2. UGT2B7

A large amount (94%) of OXC is metabolized by UGT, which shows significant differences in the activity among different ethnic groups and individuals. As an important SNP of *UGT*, the *UGT2B7* gene is located at 4q13 and is 16 kb in length containing a total of 6 exons and 5 introns, encoding 529 amino acids [228]. Ma *et al*. have conducted a statistical analysis on the relationship between the genetic polymorphism of *UGT2B7 T802C* locus and the OXC plasma concentration in Chinese patients of epilepsy [229]. Results show that the mutation rate of the *UGT2B7 T802C* locus in the selected population is 66%, which is consistent with that reported previously [230]. Furthermore, there are significant differences in the maintenance dose of OXC in patients with three genotypes at *UGT2B7 T802C* locus in the order of CC > CT > TT. Patients with the C allele require a significantly higher maintenance dose of OXC than those without the mutant allele. These results are consistent with those reported by Ma *et al*. [208]. Furthermore, results of the studies on the *UGT2B7 rs7439366* locus by Shen *et al*. suggest that the genetic polymorphism of this locus may affect the sensitivity of individuals to OXC but show no significant effect on its concentration [231]. Zhang *et al*. have reported that the mutation rate of *UGT2B7 T802C* site is 67.1% [224], which is similar to 66% as reported previously but lower than 78% in Greek population [232]. The mutation rate of *UGT2B7 T802C* gene is significantly higher in Japanese population (73.2%) than that in Caucasian population (55.1%), likely due to the ethnic and regional differences [233]. However, no significant correlation is revealed between the *UGT2B7 T802C* locus and either the OXC plasma concentration or its therapeutic efficacy in children with epilepsy, probably due to the weakened expression of the *UGT* gene in children.

In summary, it is recommended that patients with *UGT2B7* mutants be given a higher maintenance dose of OXC in order to obtain a satisfactory therapeutic effect. Used in clinical treatment of epilepsy, the dose of OXC can be individualized based on the *UGT2B7* genotype. However, due to the weakened expression of *UGT* gene in children with epilepsy, the clinical treatment of drug therapy for children cannot be completely dependent on genetype testing but in combination with closely monitoring the drug plasma concentration to ensure that the individualized dosages are reasonably formulated to avoid adverse reactions.

### 8.4. Effect of transporters on the metabolism of oxcarbazepine

ABC transporters play important roles in the metabolism of OXC *in vivo*. Studies have shown that the ABC transporters play an important role in AED tolerance [234]. ABCB1 is the first enzyme investigated in the ABC family, while OXC is the substrate of P-gp. ABCC2 is mainly distributed in the luminal (apical) membranes of both hepatocytes and the proximal tubule cells in the kidney with a small portion located in the apical membrane of polar cells such as the intestine, gallbladder epithelial cells, embryos, and endothelial cells of the blood-brain barrier. Studies have shown that the up-regulation of ABCC2 expression in brain tissue in patients with epilepsy may be related to the efficacy and distribution of the drug in the body [235].

#### 8.4.1. ABCB1

The P-gp is expressed by the ABCB subfamily member 1 transporter gene (*ABCB1*), also known as the multi-drug resistance gene 1 (*ABCB1*) [236]. To date, the *C3435T* (*rs1045642*) locus in exon 26 is the most extensively studied *ABCB1* SNP [237]. Results of a systematic review and meta-analysis show that the *ABCB1 C3435T* genetic polymorphism, particularly the TT genotype, plays important roles in treatment of refractory epilepsy [238]. Shen *et al*. have investigated the effects of *ABCB1* gene mutations on the plasma concentration of OXC and the treatment of epilepsy [231]. Results show that the *ABCB1 C3435T* genetic polymorphism is significantly associated with standardized OXC plasma concentration and its efficacy. Patients with the CC genotype show higher plasma concentration than those of CT and TT genotypes with patients of homozygous CC genotype at the *ABCB1 C3435T* locus showing the best therapeutic effect with OXC. Seo *et al*. have reported that the therapeutic efficacy of drugs varies in patients of epilepsy with different *ABCB1 C3435T* genotypes and there is no statistical difference in the plasma concentration dose ratio (C/D) of CBZ in the plasma of different genotype groups, suggesting that patients in the drug-resistant genotype group may develop drug resistance by restricting AEDs to pass through the blood-brain barrier to reach brain cells [119].

In addition to the *C3435T* locus, Yue *et al*. have analyzed the relationship between the genetic polymorphisms of *ABCB1 rs1002204* and *rs10234411* loci and the OXC plasma concentration by comparing the OXC plasma concentration dose ratio (C/D) of different genotype groups [239]. Results show that there is no significant difference in the C/D ratio among the three genotype groups, which is consistent with the hypothesis proposed previously, *i.e*., the overexpression of P-gp in the blood-brain barrier of patients caused by various factors does not affect the drug absorption of AEDs and distribution in plasma but restrict AEDs from entering brain cells, causing the difficulty for AEDs to reach effective concentrations in the brain [240–243]. These results suggest that the TT genotype at *rs1002204* and *rs10234411* may be a genetic risk factor for patients prone to drug resistance. The TT genotype may affect the brain tissue drug concentration of the patient without changing the C/D ratio of OXC in the patient<apos;>s blood, whereas leading to the drug resistance. Therefore, in order to compensate for the insufficient drug concentration in brain tissue, patients with TT genotype should be given increased doses of drugs to achieve high drug plasma concentrations.

#### 8.4.2. ABCC2

To date, studies have shown that some AEDs (*e.g*., CBZ and PHT) are the substrates of MRP2, which is involved in the transport of AEDs in the blood-brain barrier [244]. Most studies on the effects of *ABCC2* on AEDs focusing on drug resistance derive inconsistent conclusions, while studies on *ABCC2* affecting drug transport in the body are sparse [245]. Recent studies have shown that *ABCC2 G1249A* mutation decreases the transport of CBZ on the cell membrane, leading to increased adverse reactions in the central nervous system, while no correlation is identified between *ABCC2* and either the plasma concentration or the dose of CBZ [246]. OXC is a derivative of CBZ, while studies focusing on the effects of *ABCC2* on the maintenance dose and plasma concentration of OXC are currently sparse. Ma *et al*. have reported that the mutation frequencies of *ABCC2 C3972T* and *G1249A* in Chinese populations are 12.0% and 10.5%, respectively [229], slightly different from those reported in other ethnic groups (18.8% and 12.5%) [246]. Furthermore, there are significant differences in the maintenance dose of OXC in patients with three different genotypes at *ABCC2 G1249A* in the order of AA > GA > GG. Moreover, patients carrying the A allele require a significantly higher maintenance dose of OXC than patients without the mutant allele. However, Shen *et al*. have not identified the significant impact of the *G1249A* locus genetic polymorphism on the therapeutic concentration and efficacy of OXC, probably due to either insufficient samples of patients or differences in the adjustment methods of multiple factors involved in the mebabolism of OXC [231].

## 9. Levetiracetam

As one of the new generation AEDs, levetiracetam (LEV) was approved for clinical use in the 1990s, showing ideal pharmacokinetic characteristics. The LEV is not metabolized by either the liver or the cytochrome P450 enzyme system, not interacting with other AEDs but mainly excreted through kidneys [247]. Several studies have demonstrated that addition of LEV is effective and safe in the treatment of intractable or partial epilepsy. The possible functional mechanisms of LEV include: (1) LEV specifically binds to the SV2A protein to inhibit the abnormal discharge of the epileptic loop, thereby blocking the occurrence of epilepsy [248], (2) LEV inhibits the release of calcium by binding with Sri Lanka cinnamicine receptors and inositol triphosphate receptors [249], (3) LEV strengthens the inhibitory effect of the GABA circuit by preventing the decrease of GABA receptors without altering the concentration of GABA in brain tissue [250,251], and (4) LEV is not a substrate of either P-gp or MRP but can enter brain tissue through the blood-brain barrier [252,253].

The ~50 transporters of the ABC superfamily in humans are categorized into 7 subtypes, named as ABCA to ABCG, transporting multiple substrates including drugs, lipids, and ions. Three out of these transporters, *i.e*., P-gp, ABCC1, and ABCG2, are mainly related to epilepsy drug resistance [254]. P-gp is a protein in the ABC family, highly expressed on the surface of capillary endothelial cells and astrocytes of the blood-brain barrier in patients with refractory epilepsy. P-gp decomposes ATP to provide energy to pump AEDs into the blood, reducing the drug concentration in the epileptic center and failing to effectively inhibit the epileptic discharge of brain cells [255]. Several commonly used AEDs (*i.e*., PHT, PB, and CBZ) are substrates of P-gp. The ability of P-gp to efflux AEDs is associated with dosage and decreases in the order of PHT > CBZ > LTG > PB > VPA [256], LEV, and gabapentin (GBP). The *ABCB1* gene encoding P-gp is located in *C3435T* of exon 26 of chromosome 7, and contains three polymorphic genotypes CC, CT, and TT, with the up-regulation of the CC gene leading to the overexpression of P-gp [257], ultimately causing resistance to AEDs in patients with epilepsy.

Zheng *et al*. have studied the relationship between the therapeutic effect of adding LEV to the treatment of refractory epilepsy and the *ABCB1* genetic polymorphism [258]. Results show that after the testing subjects with refractory epilepsy expressing CC gene and generating P-gp are treated with addition of LEV, the seizure rate is reduced by more than 66% (*i.e*., 6 out 9 patients), while the seizure rate in the placebo group is decreased by ~14% (*i.e*., 1 out 7 patients), suggesting that LEV is not the substrate of P-gp and is capable of escaping the P-gp to enter the epileptogenic center through the blood-brain barrier. Gao *et al*. have demonstrated that the *ABCB1 C3435T* genetic polymorphism is not related to AED resistance [259], which is consistent with the results reported previously [150,260]. It is speculated that the cause of AED resistance may be related to the genetic polymorphism of other genetic sites. Furthermore, several studies have shown that *ABCB1 C3435T* genetic polymorphism may be the determining factor of AED resistance [95,119,261]. The variations in these experimental results may be caused by the limitation of sample size, inconsistent classifications of seizure types, and multi-site effects of refractory epilepsy.

In summary, it is commonly believed that the genetic polymorphism of *ABCB1* is associated with the occurrence of refractory epilepsy. However, some studies have not identified this significant association, while a large number of studies show that the overexpression of P-gp is the cause of intractable epilepsy. As a drug for the treatment of refractory epilepsy, LEV has shown satisfactory therapeutic effects and safety with its plasma concentration and efficacy not affected by *ABCB1* genetic polymorphism. This is the commonly accepted mechanism regulating the LEV passing through the blood-brain barrier and effectively acting on the epileptic center.

## 10. Summary and prospect

In recent years, genetic testing has gradually become an important support for medication in the clinical application of AEDs in the treatment of patients with epilepsy, providing therapeutic guidance for the selection of clinical AEDs. Based on the metabolic characteristics of both traditional and new AEDs, the detection of important sites of drug-metabolizing enzymes and transporter genes is important and helpful to effectively analyze and predict the response of different patients to different types of AEDs, ultimately to establish the individualized doses. For patients with epilepsy, the individualized drug delivery would facilitate the quick and accurate establishment of the treatment window, avoid drug waste, reduce economic pressure, choose the appropriate type and dosage of drugs with the least adverse reactions, and choose the types of AEDs that are not easily tolerated to maintain long-term curative effects. Therefore, the investigation and detection of genes encoding the drug-metabolizing enzymes are of significant importance to the clinical treatment of epilepsy. This subject needs to be further seriously studied and systematically summarized. In this review, we have summarized the recent advancement in our understanding of the therapeutic effects of genetic polymorphism on the plasma concentration of six types of AEDs currently commonly used in the clinical treatment of epilepsy (Table 1). Based on these studies, the clinical dosages are recommended in the individualized treatment plan of patients of epilepsy using both the traditional and newly developed AEDs.

**Table 1.** Summary of effects of genetic polymorphism on the plasma concentration of six types of antiepileptic drugs. Symbol ‘ – ’ indicates information not available. Symbols ‘↑’ and ‘↓’ indicate the increase and decrease of the drug plasma concentration, respectively

AED	Genetic polymorphism	Gene	Locus	Allele	Effect on the plasma concentration or other effects	Reference	Clinical recommendation on dosage
Valproic acid	*CYP450*	*CYP2A6*	CYP2A6*4	CYP2A6*4	↑	[31]	Decrease
		*CYP2B6*	c.516 G > T; c.785A>G	CYP2B6*6	↑	[39]	Decrease
		*CYP2C9*	c.1075A>C	CYP2C9*3	Low probability of liver toxicity	[45]	Decrease
		*CYP2C19*	c.681 G > A	CYP2C19*2	↑	[46]	Decrease
			c.636 G > A	CYP2C19*3	↑	[46]	Decrease
		*CYP2D6*	c.100C>T	CYP2D6*10	–	[54]	–
	*UGT*	*UGT1A3*	c.31 T > C	UGT1A3*2	↓	[61]	Increase
			17A>G-31 T > C-81 G > A-477A>G	UGT1A3*5	↓	[77]	Increase
		*UGT1A6*	c.541A>G	UGT1A6*2	↓	[66]	Increase
			c.552A>C			[68]	
			c.19 T > G			[69]	
		*UGT1A9*	c.98 T > C	UGT1A9*3a	–	[71]	–
			c.441C>T				
			c.1819 T > C				
			c.1888 T > C				
			c.I399C>T				
		*UGT2B7*	c.268A>G	UGT2B7*2	↓	[73,76]	Increase
			c.802C>T			[77]	
			c.211 G > T	UGT2B7*3	–	[57,73]	–
	Transporter genes	*ABCC2*	c.3972C>T; c.24C>T		↓; low therapeutic effect	[87]	Increase
			c.1249 G > A		↑	[90]	Decrease
		*ABCB1*	c.3435C>T; c.1236C>T		effective to control seizures	[84]	–
Carbamazepine	*CYP450*	*CYP3A4*	c.82266 G > A	CYP3A4*1G	↓	[103,105]	Increase
			c.608C>T		–	[103]	–
		*CYP3A5*	c.6986A>G	CYP3A5*3	↑	[109–111]	Decrease
	*UGT*	*UGT2B7*	c.802C>T	UGT2B7*2	–	[113]	–
	Transporter genes	*ABCB1*	c.3435C>T		↓	[104]	Increase
		*ABCC2*	c.24C>T; c.1249 G > A; c.3972C>T		–	[104,123]	–
Phenytoin	*CYP450*	*CYP2C9*	c.1075A>C	CYP2C9*3	↑	[136]	Decrease
		*CYP2C19*	c.681 G > A	CYP2C19*2	↑	[142,144]	Decrease
			c.636 G > A	CYP2C19*3			
	Transporter genes	*ABCB1*	c.3435C>T		effective to control seizures	[95]	–
			c.1236C>T; c.2677 G > T/A			[152]	
Lamotrigine	*UGT*	*UGT1A4*	c.142 T > G		↓; less effective to control seizures	[172,175,176]	Increase
		*UGT2B7*	c.161C>T		↑	[182]	Decrease
			c.372A>G		↓		Increase
	Transporter genes	*OCT1*	c.1222A>G		↓	[189]	Increase
			c.1022C>T		–		–
		*ABCG2*	c.421C>A		↑	[181,189]	Decrease
			c.34 G > A		–	[189]	–
		*ABCB1*	c.1236C>T		↓	[198]	Increase
			c.3435C>T; c.2677 G > T/A		–		–
Oxcarbazepine	*CYP450*	*CYP2C19*	c.636G>A	CYP2C19*3	↑	[219]	Decrease
			c.681 G > A	CYP2C19*2	–	[134]	–
	*UGT*	*UGT1A9*	c.I399C>T		Less effective to control seizures	[225]	–
		*UGT2B7*	c.802C>T	UGT2B7*2	↓	[208]	Increase
	Transporter genes	*ABCB1*	c.3435C>T		↓; less effective to control seizures	[119,231]	Increase
		*ABCC2*	c.1249 G > A		↓	[208]	Increase
Levetiracetam	Transporter genes	*ABCB1*	c.3435C>T		–	[258,259]	–

To date, the detection of drug-metabolizing genes has been widely used in the clinical treatment of epilepsy in many major hospitals in China. The patients’ DNA samples are routinely collected from the patients’ oral mucosal epithelial cells to detect the target genes, subsequently determining the selection of appropriate AEDs for the patients with low risk of adverse reactions and high level of bioavailability based on the phenotypes of different target genes. These results facilitate significantly the establishment of a unique genetic database of the Chinese population, ultimately developing the clinical guidance distinctive for the Chinese population based on the characteristics of genetic distribution. Furthermore, the detection and acquisition of a large amount of data provide strong theoretical and experimental evidence for the development and production of novel AEDs. It is reasonably expected that in the future, it is highly possible to provide detailed recommendations of the types, dosages, and frequency of administration of the personalized AEDs based on the convenient collections of the oral mucosal epithelial cells.

## 11. Conclusion

Due to its severe impact on human health, epilepsy is extensively investigated to explore its effective treatments worldwide. Although the traditional AEDs have shown serious side effects, the current treatment of epilepsy is still commonly based on the traditional AEDs. The clinical knowledge on the effects of genetic polymorphism of genes encoding drug-metabolizing enzymes on the plasma concentration of AEDs has been significantly expanded. It is increasingly imperative to summarize and conceptualize the clinical significance of recent studies on individualized therapeutic regimens. Our review summarizes solid experimental evidence to provide the clinical guidance for the applications of AEDs.

## Highlights

- Clinical significance of individualized therapeutic regimens of antiepileptic drugs
- Effects of genetic polymorphism on the plasma concentration of antiepileptic drugs
- Clinical significance of genotypes related to drug metabolism on drug dosage

Clinical guidance is provided for the therapeutic application of antiepileptic drug
